# LINE-1 retrotransposons contribute to mouse PV interneuron development

**DOI:** 10.1038/s41593-024-01650-2

**Published:** 2024-05-21

**Authors:** Gabriela O. Bodea, Juan M. Botto, Maria E. Ferreiro, Francisco J. Sanchez-Luque, Jose de los Rios Barreda, Jay Rasmussen, Muhammed A. Rahman, Laura R. Fenlon, Natasha Jansz, Carolina Gubert, Patricia Gerdes, Liviu-Gabriel Bodea, Prabha Ajjikuttira, Darwin J. Da Costa Guevara, Linda Cumner, Charles C. Bell, Peter Kozulin, Victor Billon, Santiago Morell, Marie-Jeanne H. C. Kempen, Chloe J. Love, Karabi Saha, Lucy M. Palmer, Adam D. Ewing, Dhanisha J. Jhaveri, Sandra R. Richardson, Anthony J. Hannan, Geoffrey J. Faulkner

**Affiliations:** 1 Queensland Brain Institute, University of Queensland, Brisbane, Queensland Australia; 2 Mater Research Institute - University of Queensland, TRI Building, Woolloongabba, Queensland Australia; 3 Institute of Parasitology and Biomedicine ‘López-Neyra’, Spanish National Research Council, Granada, Spain; 4 Florey Institute of Neuroscience and Mental Health, University of Melbourne, Parkville, Victoria Australia; 5 Clem Jones Centre for Ageing Dementia Research, Queensland Brain Institute, University of Queensland, Brisbane, Queensland Australia; 6 Biology Department, École Normale Supérieure Paris-Saclay, Gif-sur-Yvette, France; 7 MRC Human Genetics Unit, Institute of Genetics and Cancer, University of Edinburgh, Western General Hospital, Edinburgh, UK; 8 Department of Pharmaceutical Sciences, South Dakota State University, Brookings, SD USA; 9 Florey Department of Neuroscience and Mental Health, University of Melbourne, Parkville, Victoria Australia; 10 Present Address: Department of Genetics, University of Cambridge, Cambridge, UK

**Keywords:** Neuronal development, Genetics of the nervous system, Epigenomics, Inhibition

## Abstract

Retrotransposons are mobile DNA sequences duplicated via transcription and reverse transcription of an RNA intermediate. *Cis*-regulatory elements encoded by retrotransposons can also promote the transcription of adjacent genes. Somatic LINE-1 (L1) retrotransposon insertions have been detected in mammalian neurons. It is, however, unclear whether L1 sequences are mobile in only some neuronal lineages or therein promote neurodevelopmental gene expression. Here we report programmed L1 activation by SOX6, a transcription factor critical for parvalbumin (PV) interneuron development. Mouse PV interneurons permit L1 mobilization in vitro and in vivo, harbor unmethylated L1 promoters and express full-length L1 mRNAs and proteins. Using nanopore long-read sequencing, we identify unmethylated L1s proximal to PV interneuron genes, including a novel L1 promoter-driven *Caps2* transcript isoform that enhances neuron morphological complexity in vitro. These data highlight the contribution made by L1 *cis*-regulatory elements to PV interneuron development and transcriptome diversity, uncovered due to L1 mobility in this milieu.

## Abstract (web summary)

LINE-1 retrotransposons are a type of mobile DNA element normally repressed in the body. Here the authors show that LINE-1 sequences can jump in mouse parvalbumin interneurons and also promote the transcription of key parvalbumin interneuron genes.

## Main

Brain activity is governed by complex and coordinated interactions between neurons diverse in function and developmental history. Long interspersed element 1 (LINE-1 or L1) retrotransposons compose nearly 20% of the human and mouse genomes^1^. To retrotranspose, or duplicate, an L1 sequence initiates the transcription of a full-length (>6 kbp) messenger RNA from its canonical 5′ untranslated region (UTR) promoter. This mRNA encodes two proteins, denoted ORF1p and ORF2p, that bind the L1 mRNA and facilitate its retrotransposition by target-primed reverse transcription^2–5^. Heritable L1 insertions principally arise in the early embryo or primordial germ cells and can be pathogenic^6–10^. Somatic L1 retrotransposition can also occur in the brain and cause neuronal genome mosaicism, as first revealed by L1 retrotransposition reporter assays^11–14^ and later confirmed by single-cell genomic analyses of pan-neuronal (NeuN^+^) populations^15–18^. Whether endogenous L1 retrotransposition is more common in some neuronal lineages than in others, or impacts neurobiology, is unclear.

In addition to full-length L1 mRNA production, the L1 5′UTR can bidirectionally promote the formation of chimeric RNAs with adjacent genes, potentially leading to gene expression in novel spatiotemporal contexts^17,19–22^. L1 sequences are therefore, like many retrotransposons, a genomic reservoir of *cis*-regulatory element innovation^23^. DNA methylation and histone modifications repress L1 5′UTR promoter activity and vary among brain regions and neuron subtypes^17,24–31^. Transcription factors, including YY1 and members of the SOX family, are also known to regulate the L1 5′UTR^11,12,17,25,29,32–34^. For example, SOX2 represses L1 transcription in pluripotent and neural stem cells^11,12^. SOX2 downregulation during neurodifferentiation is proposed to facilitate L1 retrotransposition^11,12^. It is unclear whether other SOX proteins, which can be tissue and cell lineage specific^35^, activate or repress the L1 5′UTR in the absence of SOX2. The combined epigenomic landscape and transcription factor repertoire of each neuronal lineage could thus create niches for L1 sequences to mobilize or upregulate nearby genes.

In this Article, we reveal L1 activation in the mouse parvalbumin (PV) interneuron lineage. These inhibitory interneurons are essential for circuit plasticity, processing of sensory information and memory consolidation^36,37^. Crucially, PV interneuron development depends on a SOX6-directed transcriptional program^38–40^. Using a range of orthogonal in vivo and in vitro experimental approaches, we find that PV interneurons permit L1 mRNA expression and retrotransposition, stimulated by SOX6. Unmethylated L1 promoters produce chimeric protein-coding RNAs with adjacent genes that, as we highlight in the *Caps2* locus, hold substantial potential to alter PV interneuron morphological complexity and function.

## Results

### PV interneurons support L1 retrotransposition

**Fig. 1:**
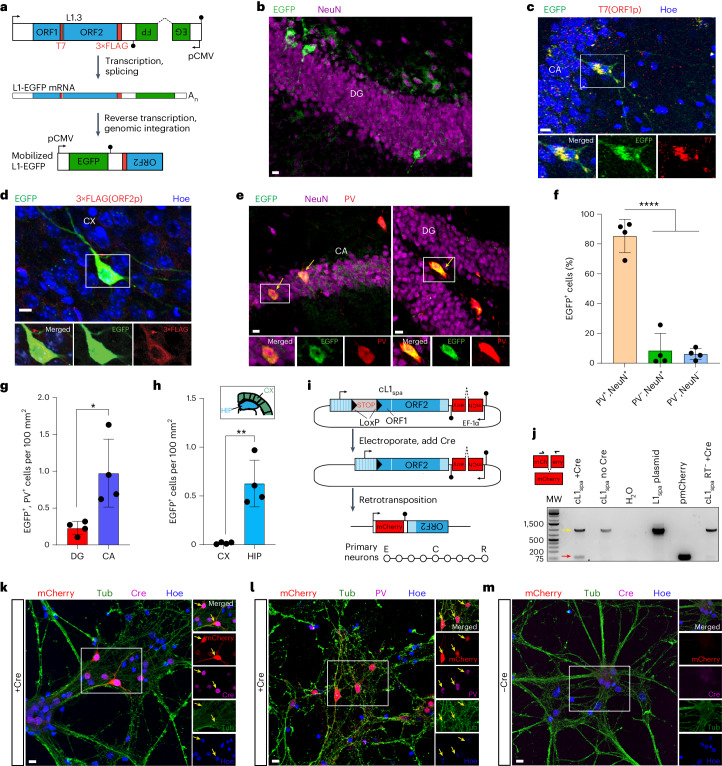
L1 retrotransposition in PV interneurons. **a**, L1-EGFP reporter schematic. A mobile human L1 (L1.3) is expressed from its native promoter, harbors epitope tagged ORF1 (T7) and ORF2 (3×FLAG) sequences, and carries an EGFP indicator cassette. The EGFP is antisense to the L1, incorporates a ɣ-globin intron in the same orientation as the L1 and is terminated by a polyadenylation signal (filled black lollipop). L1-EGFP retrotransposition removes the ɣ-globin intron, enabling EGFP expression. **b**, Example EGFP^+^ cells detected in the hippocampus. **c**, A representative confocal image of ORF1p (T7) immunostaining of L1-EGFP adult mouse brain. The image insets show a selected cell in merged and single channels for EGFP and ORF1p. **d**, As for **c**, except for ORF2p (3×FLAG) in cortex (CX). **e**, EGFP and PV immunostaining of L1-EGFP mouse coronal hippocampus sections. The yellow arrows indicate EGFP^+^ neurons. **f**, The percentage of hippocampal EGFP^+^ cells colocalized with NeuN and PV. ****P = 0.0001, one-way ANOVA with Tukey’s post-hoc test. **g**, The distribution of EGFP^+^/PV^+^ cells counted in the main hippocampal substructures. *P = 0.02, two-tailed *t*-test. **h**, EGFP^+^ cell counts in cortex and hippocampus (HIP). **P = 0.002, two-tailed *t*-test. Note: **f**–**h** represent data as mean ± s.d., and *N*(mice) = 4. **i**, cL1_spa_ reporter schematic. A mobile mouse L1 (L1_spa_) is expressed from its native monomeric 5′UTR, harbors a LoxP-Stop-LoxP cassette and carries an mCherry indicator cassette. Cre-Lox recombination removes the Stop, enabling L1_spa_ transcription and retrotransposition. Primary neurons were electroporated (E) with cL1_spa_, then transduced with a Cre (C) lentivirus 4 days later, and results (R) were analyzed 8 days post-electroporation. **j**, mCherry splice junction PCR assay. The black arrows above the mCherry cassette (left) indicate oligo positions. 994 bp product: unspliced, 92 bp product: spliced. Gel lanes (left to right): molecular weight (MW) ladder, primary neurons electroporated with cL1_spa_ with or without Cre, nontemplate control, L1_spa_ plasmid positive control, pmCherry plasmid positive and cL1_spa_ RT^−^ mutant negative control. The red and yellow arrows indicate the expected sizes of the spliced and unspliced DNA products, respectively. **k**, Representative confocal images of mCherry immunostaining in primary neurons upon Cre addition. The image insets show cells in merged (top) and single channels for mCherry, Cre, Tub and Hoechst (Hoe) (nuclei). The yellow arrows indicate mCherry^+^ neurons. **l**, As in **k** but showing mCherry and PV colocalization. **m**, As in **k** except showing mCherry immunostaining without Cre. Scale bars, 10 µm (**b**–**e** and **k**–**m**). Source data

**Extended Data Fig. 1:**
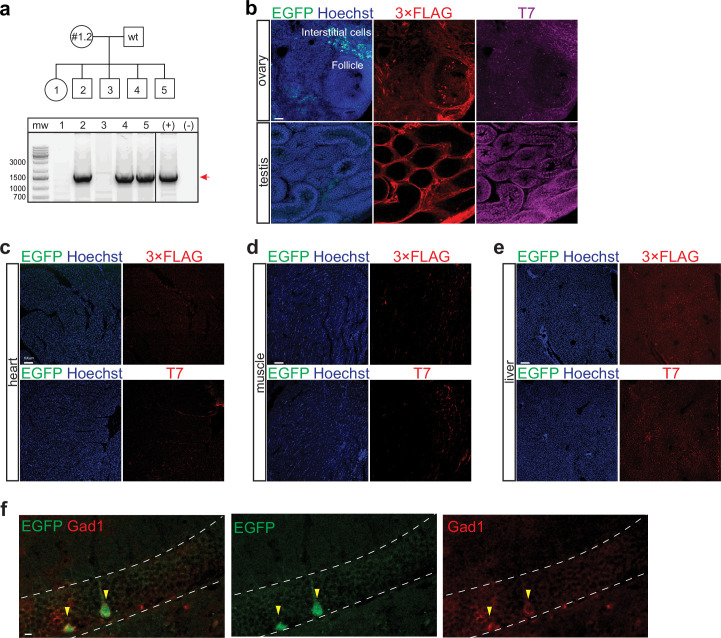
Assessment of L1 retrotransposition in brain and non-brain tissues of L1-EGFP animals. **a**, EGFP cassette genotyping PCR results for the offspring of founder animal #1.2. Circles and squares represent female and male mice, respectively. The 1.7kbp PCR product (red arrow) corresponds to the integrated intron-containing EGFP indicator cassette. Gel labels are as follows: mw, molecular weight (ladder); 1-5, transgenic offspring littermates; +, EGFP positive control plasmid DNA; -, H_2_0. Offspring 2, 4 and 5 carried the L1-EGFP transgene. **b**, Ovary and testis **c**, heart **d**, muscle and **e**, liver tissues of adult L1-EGFP mice were immunostained for EGFP and L1 proteins (T7-tagged ORF1p and 3×FLAG-tagged ORF2p). EGFP^+^ cells were observed in the interstitial cells of the ovaries but not in other tissues. DNA was stained with Hoechst dye (blue). **f**, Representative maximum intensity projection confocal image of a coronal hippocampus section from a transgenic L1-EGFP animal showing immunostaining for EGFP (green) and the interneuron marker Gad1 (red). Yellow arrowheads indicate EGFP^+^/Gad1^+^ neurons in DG. The image is presented as merged and single channels for EGFP and Gad1. Scale bar: 100 µm. Source data

To resolve the spatial and cell type specificity of somatic L1 mobility in vivo, we generated a transgenic L1-EGFP mouse line (where EGFP is enhanced green fluorescent protein; Fig. 1a and Extended Data Fig. 1a) harboring a retrotransposition reporter based on L1.3, a highly mobile human L1 element^41,42^. L1.3 was expressed from its native promoter and incorporated T7 and 3×FLAG epitope tags on ORF1p and ORF2p, respectively (Fig. 1a). Immunofluorescence revealed EGFP^+^ neurons (Fig. 1b). In agreement with prior experiments^11^, nearly all EGFP^+^ cells were found in the brain, apart from occasional EGFP^+^ ovarian interstitial cells (Extended Data Fig. 1b–e). Tagged ORF1p and ORF2p expression was observed in EGFP^+^ neurons, indicating that the L1 protein machinery coincided with retrotransposition (Fig. 1c,d). Guided by their location and morphology, we hypothesized that the EGFP^+^ cells were predominantly PV interneurons. Immunostaining indicated that 85.4% of EGFP^+^ hippocampal cells were PV interneurons, on average (Fig. 1e,f). PV^+^/EGFP^+^ neurons were found throughout the hippocampal dentate gyrus (DG) and cornu ammonis regions 1–3 (CA1–3, referred to here as CA; Fig. 1g) but were infrequent in the cortex (Fig. 1h). EGFP^+^ cells also expressed Gad1 (Extended Data Fig. 1f), another inhibitory interneuron marker^43^. L1-EGFP retrotransposition was thus most common in the PV interneuron lineage.

**Extended Data Fig. 2:**
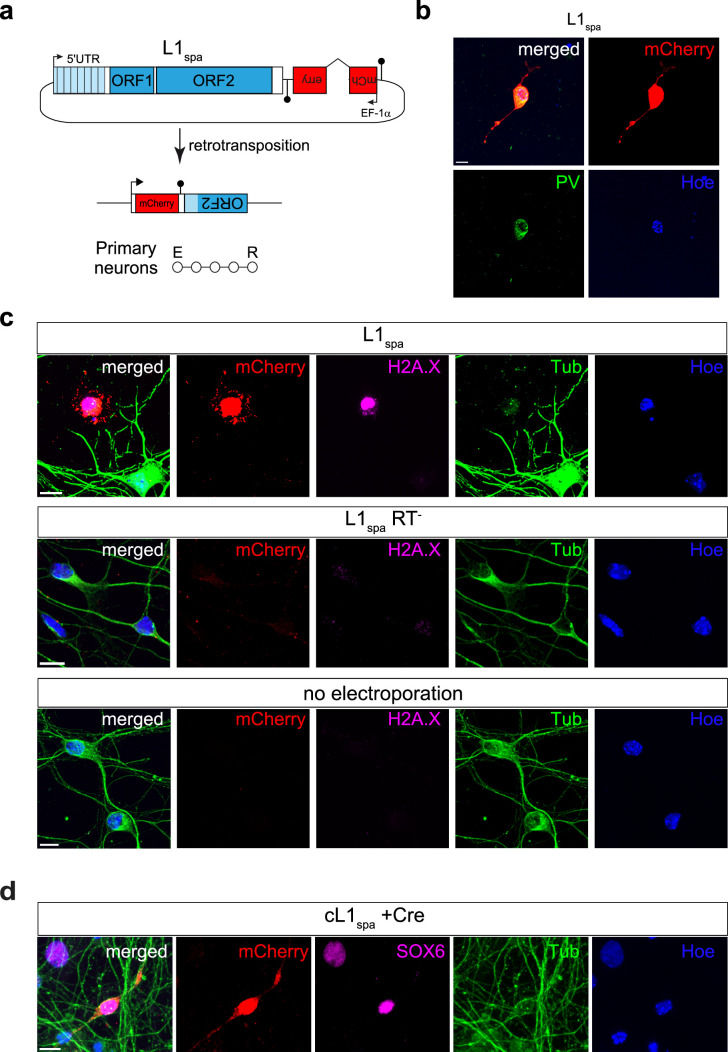
Retrotransposition of an engineered mouse L1 delivered to cultured neurons. **a**, Primary neurons were electroporated with an L1_spa_ reporter construct driven by its native promoter and carrying an mCherry indicator cassette. The mCherry is antisense to the L1, incorporates a ɣ-globin intron in the same orientation as the L1, and is terminated by a polyadenylation signal (filled black lollipop). Retrotransposition removes the ɣ-globin intron, enabling mCherry expression. Experimental timeline is shown below the schematic. Circles represent days in culture, E: electroporation, R: analysis. **b**, Example of a degenerating neuron expressing mCherry (red) and parvalbumin (PV, green). **c**, Representative confocal images of H2A.X (magenta) immunostaining of neurons (Tub, green) shows DNA breaks in mCherry neurons after L1_spa_ electroporation. Immunostaining of L1_spa_ RT^−^ mutant (retrotransposition negative control) and no electroporation controls are shown in the panels below. **d**, Representative confocal images of cL1_spa_ reporter electroporation showing mCherry (red) and SOX6 (magenta) colocalization in primary neurons upon Cre addition. Note: scale bars in (b-d) indicate 10 µm.

**Extended Data Fig. 3:**
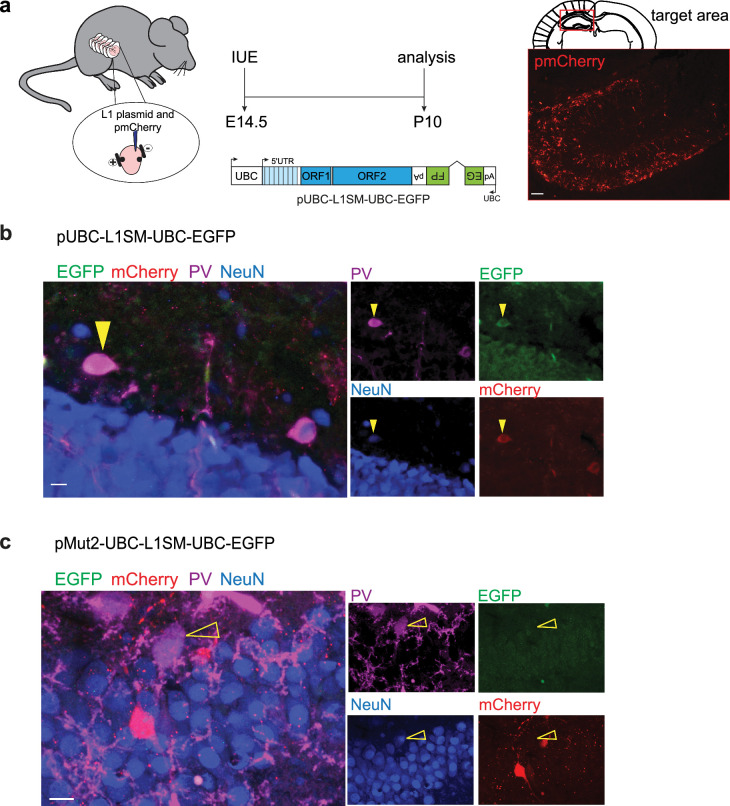
Retrotransposition of an engineered mouse L1 delivered *in utero.* **a**, Schematic representation of L1-EGFP reporter *in utero* electroporation (IUE). A coronal view of electrode positioning is shown at left. Embryos were co-injected with pmCherry (where a CMV promoter controls mCherry expression) and a second plasmid, carrying a mouse L1 tagged with an EGFP indicator cassette (pUBC-L1SM-UBC-EGFP), into the left hemisphere. As a negative control, embryos were co-injected with pmCherry and a disabled L1 reporter plasmid (pMut2-UBC-L1SM-UBC-EGFP) into the right hemisphere. The red inset, shown at right, displays a coronal section of an electroporated mouse brain with pmCherry visible in the targeted hippocampal region. Scale bar indicates 100 µm. IUE was performed on embryonic day (E)14.5. Embryos were born and then sacrificed at postnatal day (P)10. Note: UBC-L1SM-UBC-EGFP consists of a heterologous UBC promoter driving expression of a synthetic mouse L1 T_F_ (L1SM) containing a native L1 monomeric 5′UTR promoter, codon-optimized ORF1 and ORF2 sequences, the 5′ part of the L1 3′UTR, and an intron-interrupted EGFP indicator cassette with its own UBC promoter and polyadenylation signal (pA). In this system, a cell becomes EGFP positive only when the L1-EGFP mRNA is transcribed, spliced, reverse transcribed and integrated into the genome, allowing a functional EGFP to be expressed from its UBC promoter. Mut2-UBC-L1SM-UBC-EGFP is the same as UBC-L1SM-UBC-EGFP, except it carries mutations known to disable ORF2p reverse transcriptase and endonuclease activities. **b**, Example maximum intensity projection confocal image of a hippocampus section from an embryo electroporated with UBC-L1SM-UBC-EGFP. A PV^+^ (magenta), mCherry^+^ (red), NeuN^+^ (blue), EGFP^+^ cell is indicated with a yellow arrowhead. **c**, No EGFP^+^ cells were found for the retrotransposition-incompetent Mut2-UBC-L1SM-UBC-EGFP plasmid. An empty arrowhead points to a PV^+^, NeuN^+^, mCherry^−^, EGFP^−^ cell. Note: scale bars in (a) and (b) indicate 10 µm.

As an orthogonal approach, we electroporated cultured primary mouse neurons with an L1-mCherry retrotransposition reporter based on L1_spa_, a mobile mouse L1 expressed from its native monomeric 5′UTR promoter^44–46^. While we observed PV^+^/mCherry^+^ cells (Extended Data Fig. 2a,b), the vast majority of mCherry^+^ cells died, an outcome we did not observe for an L1_spa_ ORF2p reverse transcriptase (RT) mutant reporter (Extended Data Fig. 2c). A Cre-LoxP conditional version of the L1-mCherry reporter, which we called cL1_spa_ (Fig. 1i), circumvented this apparent toxicity and retrotransposed in primary neurons (Fig. 1j–m). Nearly all present PV^+^ cells were also mCherry^+^ (Fig. 1l). Retrotransposition did not occur in the absence of Cre (Fig. 1m) or using an cL1_spa_ ORF2p RT mutant (Fig. 1j). As a complementary approach, we used in utero electroporation to deliver to the embryonic hippocampus a codon-optimized synthetic L1_spa_^47^ bearing an EGFP reporter (Extended Data Fig. 3a). We observed occasional hippocampal EGFP^+^/PV^+^ neurons in neonates (Extended Data Fig. 3b). No EGFP^+^ cells were present when the reporter, with disabled ORF2p endonuclease and RT activities^47^, was electroporated into the contralateral hemisphere (Extended Data Fig. 3c). Disparate mouse and human L1 reporters delivered by distinct methods thus retrotransposed in the PV interneuron lineage in vivo and in vitro.

### Endogenous L1 expression in PV interneurons

**Fig. 2:**
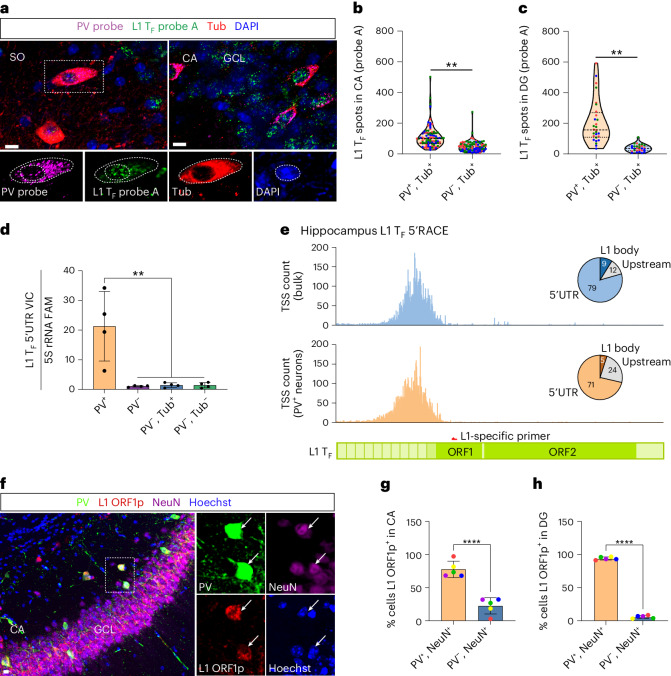
L1 mRNA and ORF1p are abundant in PV interneurons. **a**, Representative maximum intensity projection (MIP) confocal image of a coronal hippocampus section showing L1 T_F_ (green) and PV (magenta) transcripts detected by RNA FISH, Tub, (red) immunohistochemistry and DAPI staining (blue). The image insets show higher magnification of a selected PV^+^ neuron (dashed rectangle). The dashed lines in the image insets demark nuclear and cellular boundaries defined for PV and L1 mRNA quantification. Scale bars, 10 μm. GCL, granular cell layer; SO, stratum oriens. **b**, L1 T_F_ RNA FISH spot (puncta) count per cell in CA PV^+^/Tub^+^ and PV^−^/Tub^+^ neurons. **P = 0.009, *n*(cells per mouse) = 29-31, *N*(mice) = 3. Cells from different mice are color coded. Dashed line, median; dotted lines, quartiles. **c**, As for **b**, except for DG. **P = 0.009, *n* = 9–10, *N* = 3. **d**, Multiplexed TaqMan qPCR measuring mRNA abundance of the L1 T_F_ monomeric 5′UTR (VIC channel) relative to 5S rRNA (FAM channel) in PV^+^, PV^−^, PV^−^/Tub^+^ and PV^−^/Tub^−^ cell populations. Cells were sorted from pooled neonate (P0) litter hippocampi. **P = 0.0022, *N* = 4 litters (one-way ANOVA with Tukey’s post-hoc test). **e**, TSS usage within full-length L1 T_F_ copies, detected by L1-specific 5′RACE. RNA was obtained from bulk (blue, top) and sorted PV^+^ hippocampal cells (orange, bottom). The pie charts indicate the percentages of L1 T_F_ mRNAs initiating at upstream TSSs, or TSSs in the L1 T_F_ 5′UTR or body (ORF1). The L1 T_F_ diagram provided underneath indicates the position of the L1-specific primer used for 5′RACE. **f**, Endogenous ORF1p expression in PV^+^ neurons. An MIP confocal image of a hippocampus coronal section showing PV (green), ORF1p (red) and NeuN (magenta) colocalization. The insets show a higher-magnification view of a selected PV^+^/ORF1p^+^/NeuN^+^ neuron. Hoechst stains nuclei. Scale bar, 10 μm. **g**, The percentages of PV^+^/NeuN^+^ and PV^−^/NeuN^+^ neurons expressing ORF1p in hippocampus CA (regions 1–3). ****P = 0.0001, *n* = 1,017 average cells per mouse, *N* = 5. **h**, As for **g**, except in DG. ****P = 0.0001, *n* = 719, *N* = 5. Note: significance testing in **b**, **c**, **g** and **h** was via two-tailed *t*-test, comparing animal or litter mean values. Data in **d**, **g** and **h** are represented as mean ± s.d. Source data

**Extended Data Fig. 4:**
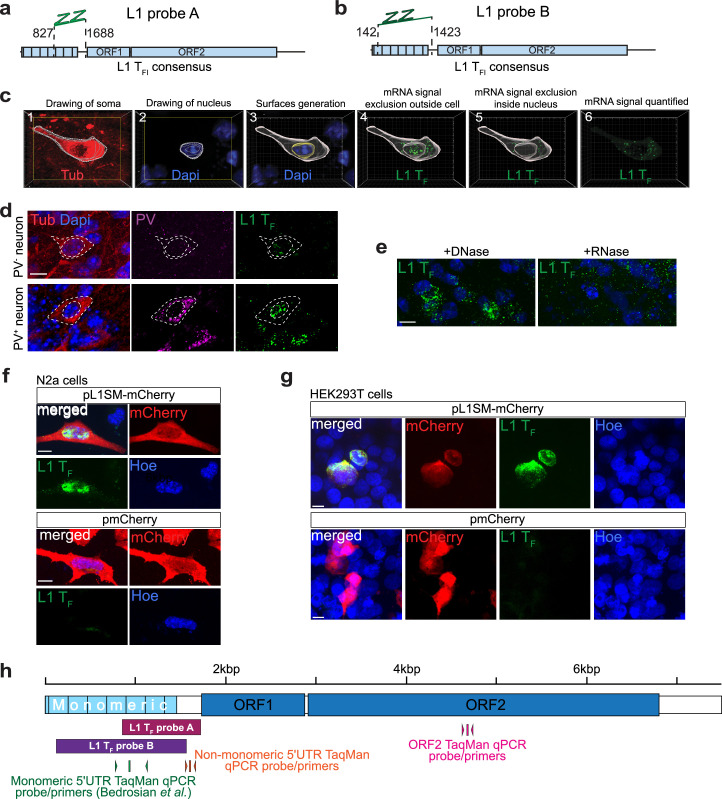
Single-molecule L1 T_F_ RNA FISH experimental design. **a**, L1 T_F_ RNAscope probe A consisted of 20 ‘ZZ’ oligo pairs targeting the L1 T_F_ 5ʹUTR monomeric and non-monomeric region (consensus positions 827 to 1688). **b**, L1 T_F_ probe B was composed of 17 ZZ oligo pairs and targeted the L1 T_F_ 5ʹUTR monomeric region (positions 142 to 1423). **c**, Imaris analysis performed on Z-stack images of L1 T_F_ and PV RNA FISH coronal hippocampus sections immunostained for Tub. Imaris workflow: 1: neuron identification, based on cytoplasmic Tub (red) fluorescence, and cell volume drawing, 2: nucleus definition by DAPI (blue) staining followed by drawing of nuclear volume, 3: delineation of cell and nucleus surfaces, 4: cell surface masking to eliminate voxels outside the cell, 5: nucleus surface masking to exclude voxels inside the nucleus, and 6: L1 T_F_ mRNA (green) fluorescence signal within the defined cytoplasmic volume. **d**, Maximum intensity projection confocal images of hippocampal sections showing L1 T_F_ probe A (green) and PV (magenta) RNA FISH, and Tub (red) immunohistochemistry. Dashed lines demark nuclear and cellular boundaries defined for PV and L1 mRNA quantification. Selected PV^−^ and PV^+^ neurons are shown top and bottom, respectively. **e**, Confocal images of hippocampal sections treated with either DNase or RNase and subsequently processed for RNAscope using the L1 T_F_ probe A (green). **f**, Confocal images of N2a cells transfected with either mouse L1 construct (pL1SM-mCherry) or control (pmCherry) showing specificity of L1 T_F_ RNA FISH probe A (green) signal in cells expressing the L1 construct. **g**, As for (f), except performed on HEK293T cells and showing specificity of L1 T_F_ RNA FISH probe B (green) in cells expressing the L1 construct. **h**, Schematic showing L1 T_F_ sequences assayed by RNA FISH and TaqMan qPCR. Scale bar for (d-g): 10 μm.

**Extended Data Fig. 5:**
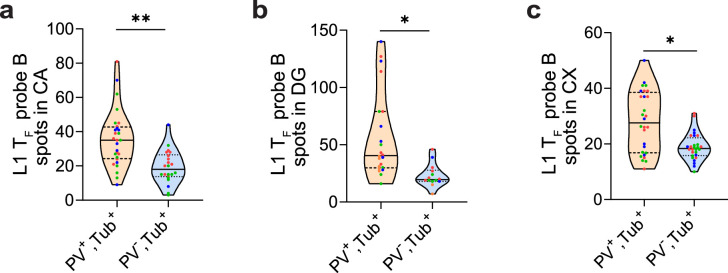
Additional L1 T_F_ RNA FISH data for hippocampus and cortex. **a-c**, Number of L1 T_F_ RNA FISH (probe B) spots in PV^+^/Tub^+^ neurons (orange plot) and PV^−^/Tub^+^ neurons (blue plot) in CA (a), DG (b) and CX (c). **P = 0.003, *P = 0.03 and *P = 0.01 for (a), (b) and (c), respectively. Two-tailed t test comparing animal means, *n*(cells)=2-10, *N*(mice)=3-4. Solid line: median. Dashed lines: quartiles. Source data

**Extended Data Fig. 6:**
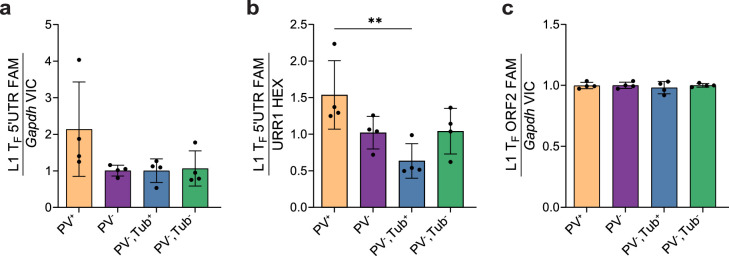
Elevated L1 transcription in PV interneurons. **a**, Multiplexed TaqMan qPCR measuring mRNA abundance of the L1 T_F_ non-monomeric 5ʹUTR sequence (FAM channel) relative to *Gapdh* (VIC channel) in sorted PV^+^, PV^−^, PV^−^/Tub^+^ and PV^−^/Tub^−^cells. Cells were sorted from pooled neonate litter hippocampi. *N* = 4 litters. **b**, As for (a), except relative to URR1 repetitive DNA (HEX channel). *P = 0.015. **c**, As for (a), except measuring L1 T_F_ ORF2 (FAM channel) relative to *Gapdh* (VIC channel). Data are represented as mean ± SD. Significance values were calculated via one-way ANOVA with Tukey’s post-hoc test. Source data

**Extended Data Fig. 7:**
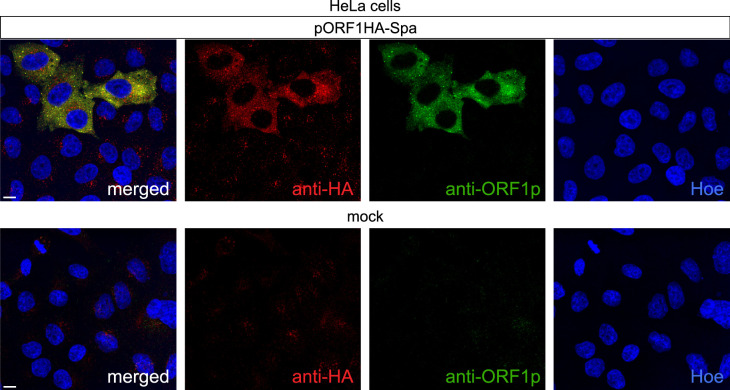
L1 ORF1p antibody specificity. Confocal images of HeLa cells transfected with either a HA-tagged ORF1p (L1_spa_) expression vector or with transfection reagents alone (mock), showing specificity of the ORF1p antibody (green) in human cells expressing mouse ORF1p. Scale bar 10 μm.

We next measured endogenous L1 expression in PV interneurons. First, we designed a custom single-molecule RNA fluorescence in situ hybridization (FISH) probe against the monomeric 5′UTR of the mouse L1 T_F_ subfamily^8,34,44^ (Extended Data Fig. 4a). With multiplexed RNA FISH, we counted cytoplasmic L1 and PV mRNA puncta in adult β-tubulin (Tub)-immunostained neurons (Fig. 2a and Extended Data Fig. 4c,d). L1 T_F_ transcription was significantly higher in PV^+^ neurons, compared to PV^−^ neurons, in CA (*P* = 0.009; Fig. 2b) and DG (*P* = 0.009; Fig. 2c). A second L1 T_F_ 5′UTR RNA FISH probe (Extended Data Fig. 4b) also showed L1 mRNA enrichment in hippocampal and cortical PV^+^ neurons (Extended Data Fig. 5). Control experiments conducted in mouse and human cells confirmed the L1 T_F_ RNA FISH probes did not detect DNA (Extended Data Fig. 4e) and were specific to L1 T_F_ mRNA (Extended Data Fig. 4f,g). Second, we used TaqMan quantitative polymerase chain reaction (qPCR) to measure L1 expression in hippocampal PV^+^ neurons and PV^−^ populations (PV^−^, PV^−^/Tub^+^ and PV^−^/Tub^−^) sorted from pooled neonate littermates (Supplementary Fig. 1). Three qPCR primer–probe combinations (Extended Data Fig. 4h) detecting the L1 T_F_ 5′UTR each indicated higher expression in PV^+^ neurons than in PV^−^ cells (Fig. 2d and Extended Data Fig. 6a,b). By contrast, qPCR targeting the L1 T_F_ ORF2 region, expected to mainly detect immobile 5′ truncated L1s incorporated in other cellular mRNAs, showed no difference between PV^+^ and PV^−^ cells (Extended Data Fig. 6c). Third, 5′RACE (rapid amplification of cDNA ends) upon bulk adult hippocampus or sorted neonate PV interneurons indicated the predominant transcription start sites (TSSs) of full-length L1 mRNAs initiated within L1 T_F_ family 5′UTR sequences (Fig. 2e). The fraction of L1 T_F_ mRNAs >6 kbp long recovered by this assay was also higher in the sorted PV interneurons (76.0%) than in the bulk hippocampus (63.8%). Fourth, immunostaining using an antibody specific to mouse L1 ORF1p (Extended Data Fig. 7) indicated that ORF1p expression (Fig. 2f) was significantly (*P* = 0.0001) higher in PV^+^ neurons than PV^−^ neurons, in CA (Fig. 2g) and DG (Fig. 2h). Finally, given that environmental stimuli can alter neural circuits involving PV interneurons^43^, we analyzed L1 activity using L1 ORF1p immunostaining and L1 T_F_ RNA FISH in adult mice housed in standard, voluntary exercise and enriched environments, and observed no consistent differences among these experimental groups (Supplementary Figs. 2 and 3). We concluded that PV interneuron enrichment for L1 T_F_ mRNA and protein expression was robust yet not significantly impacted by exercise or environmental enrichment.

### SOX6 stimulates L1 transcription

**Fig. 3:**
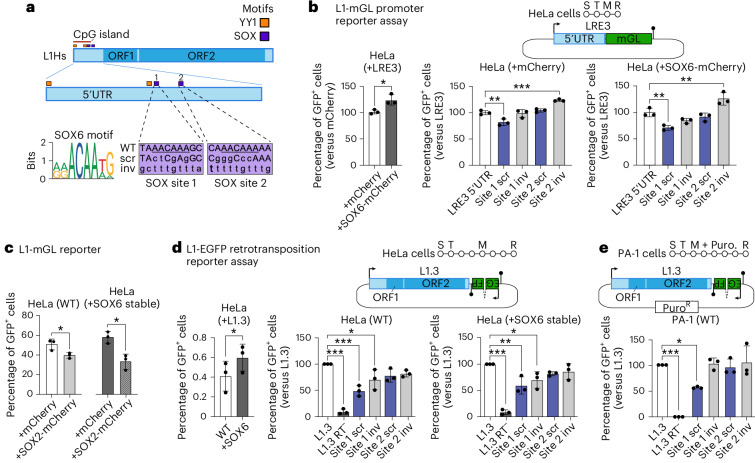
SOX motif-dependent L1 activation by SOX6. **a**, L1Hs schematic. 5′UTR embedded YY1- (orange) and SOX-binding (purple) sites are shown, with the latter numbered 1 and 2 and corresponding to L1Hs positions +470 to +477 and +570 to +577, respectively. These SOX motifs were scrambled (scr) or inverted (inv) in our L1 reporter assays. Site 1 more closely matched the JASPAR SOX6 binding site motif. **b**, L1 promoter assay. The native 5′UTR of the highly mobile L1Hs element LRE3 (top) was used to promote mGreenLantern (mGL) expression (S, seeding; T, transfection; M, change of medium; R, result analysis; filled lollipop, polyadenylation signal). The promoter strength is measured as the percentage of GFP^+^ sorted cells. LRE3 5′UTR plasmids, including those with scrambled or inverted SOX motifs, were cotransfected into HeLa cells with mCherry (middle) or SOX6-mCherry (right) expression vectors, with a higher percentage of GFP^+^ cells observed in the latter experiment (left). **c**, The reporter from **b** was cotransfected with mCherry or SOX2-mCherry expression vectors into wild-type (WT) and SOX6 stably overexpressing HeLa cells. **d**, L1 retrotransposition assay. The assay design (top) shows a highly mobile L1Hs element, L1.3 expressed from its native promoter (black arrow) and tagged with an EGFP cassette activated upon retrotransposition and driven by a CBh promoter (S, seeding; T, transfection; M, change of medium; R, result analysis; filled lollipop, polyadenylation signal). The retrotransposition efficiency is measured as the percentage of GFP^+^ sorted cells. Plasmids included positive (L1.3) and negative (L1.3 RT^−^, D702A mutant) controls and L1.3 sequences with scrambled or inverted SOX motifs. Each element was assayed in WT (middle) and SOX6 stably overexpressing HeLa cells (right), with a higher percentage of GFP^+^ cells observed in the latter experiment (left). **e**, As for **d**, except conducted in WT PA-1 cells using a CMV promoter-driven EGFP cassette and cells were selected for puromycin resistance (Puro^R^). Note: histogram data in **b**–**e** are represented as mean ± s.d. with *n* = 3 biological replicates. Significance testing in **b**, **d** and **e** was via one-way ANOVA against the corresponding positive control (LRE3 5′UTR or L1.3) with Dunnett’s multiple comparison test (middle, right or only panel) or two-tailed *t*-test (left). Significance testing in **c** was via two-tailed *t*-test. *P < 0.05, **P < 0.01, ***P < 0.001. Source data

**Extended Data Fig. 8:**
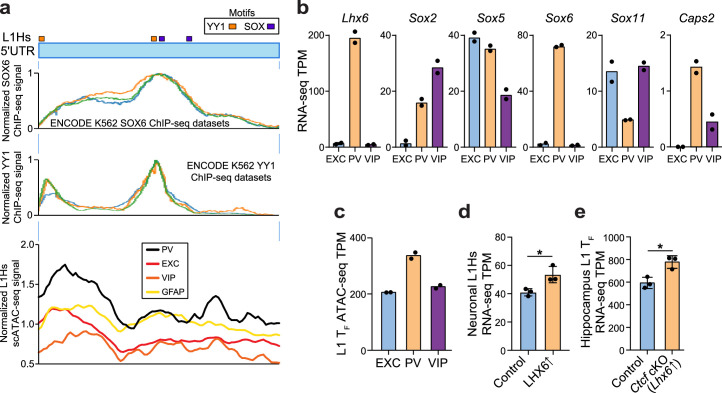
L1 activation by the LHX6/SOX6 transcriptional program. **a**, The L1Hs 5ʹUTR, with YY1- (orange) and SOX-binding (purple) sites marked, is shown above MapRRCon^48^ profiles of ENCODE K562 SOX6 and YY1 ChIP-seq binding and chromatin accessibility profiles in human hippocampal tissue as measured by scATAC-seq^49^. Cells were grouped based on selected accessible genes to define neural cell populations (PV: parvalbumin inhibitory interneuron, EXC: excitatory neurons, VIP: vasoactive intestinal polypeptide-expressing inhibitory interneurons, GFAP: glia). **b**, *Lhx6*, *Sox2*, *Sox5*, *Sox6*, *Sox11* and *Caps2* expression in excitatory (EXC) pyramidal neuron, PV interneuron and VIP interneuron cortex populations defined by Mo et al.^24^, measured by RNA-seq tags per million (TPM). *N* = 2. **c**, Proportion of ATAC-seq reads aligned to peaks associated with full-length L1 T_F_ copies in neuronal populations defined by Mo et al. *N* = 2. **d**, L1Hs subfamily expression measured by RNA-seq TPM in neurons derived via *in vitro* differentiation of induced pluripotent stem cells^50^, with (LHX6↑) and without (control) LHX6 overexpression. *P = 0.03, two-tailed t test, *N* = 3. **e**, L1 T_F_ family expression measured by RNA-seq TPM in bulk hippocampus^51^ of animals with (*Ctcf* cKO) and without (control) conditional knockout of *Ctcf* and associated induction of *Lhx6* expression. *P = 0.01, two-tailed t test, *N* = 3. Note: histogram data in (d,e) are represented as mean ± SD. Source data

**Extended Data Fig. 9:**
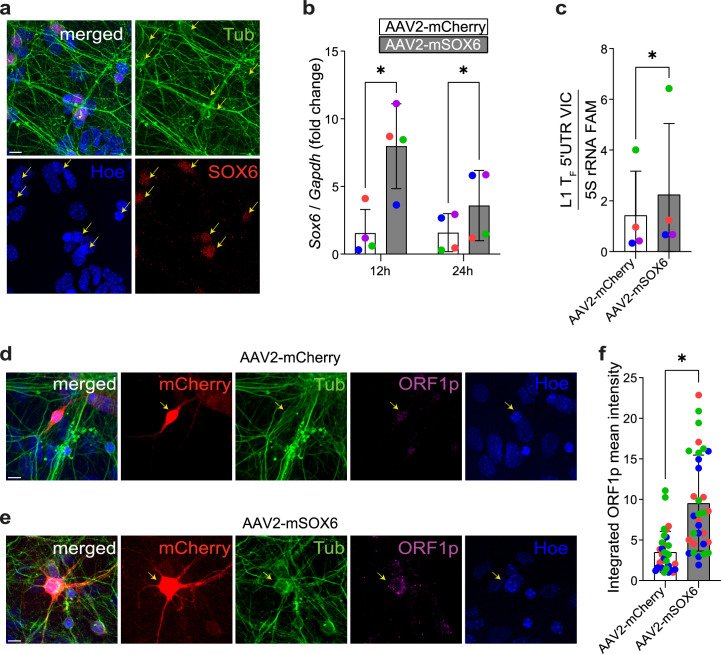
SOX6 overexpression is associated with increased L1 transcription and ORF1p expression in primary neuronal cultures. **a**, Confocal images of mouse primary neuronal cultures showing SOX6 (red) expression in Tub^+^ (green) neurons. **b**, Primary neuronal cultures after 5 days *in vitro* were transiently transduced with AAV2 vectors carrying either mCherry or a mouse SOX6 construct (mSOX6-T2A-mCherry) under the control of a CBh promoter. qPCR analysis at 12 and 24 h post-transduction revealed a significant increase in SOX6 mRNA expression in mSOX6-T2A-mCherry transduced cultures compared to mCherry controls. *P < 0.03, two-way ANOVA with Tukey’s multiple comparison test, *N* = 4. **c**, TaqMan qPCR measuring abundance of the L1 T_F_ mRNA monomeric 5ʹUTR (VIC channel) relative to 5 S rRNA (FAM channel) in mCherry versus mSOX6-T2A-mCherry 24 h post-transduction. *P = 0.012, paired two-tailed t test, *N* = 4. **d**, Images showing L1 ORF1p (magenta), mCherry (red) and Tub (green) immunostaining in neurons 72 h post-transduction with AAV2-mCherry. **e**, As per (d), except for mSOX6-T2A-mCherry. **f**, Analysis of L1 ORF1p intensity in mCherry^+^ neurons shows significantly more ORF1p expression in mSOX6-T2A-mCherry transduced neurons than in mCherry controls. *P = 0.015, two-tailed t test, *N* = 3 transductions, *n* = 10-12 cells quantified per experiment. Note: data in (b), (c) and (f) are from independent experiments, with replicates color coded and represented as mean ± SD. Scale bar: 10 μm. Source data

SOX transcription factors can regulate L1 promoters^11,12,33^. For instance, the youngest human-specific L1 (L1Hs) subfamily contains two consensus SOX motifs (first site: +470 to +477, second site: +570 to +577) known to bind SOX proteins (Fig. 3a), including SRY, SOX2 and SOX11 (refs. ^11,12,33^). Given that L1Hs transcriptional repression by SOX2 is generally released upon neural stem cell differentiation^11,12,35^, we considered whether SOX2 is replaced by another SOX protein in binding the L1 5′UTR in the committed PV interneuron lineage. Notably, SOX6 coordinates a major transcriptional program of the embryonic and adult brain downstream of LHX6, is necessary for PV interneuron development^38,39^, can bind the first SOX site (Extended Data Fig. 8a)^48^ and, of the major hippocampal neuron types, is the only SOX protein specific to PV interneurons (Extended Data Fig. 8b). cL1_spa_ reporter assays conducted in primary mouse neurons (Extended Data Fig. 2d), analyses of human and mouse assay for transposase-accessible chromatin with sequencing (ATAC-seq) datasets^24,49^ (Extended Data Fig. 8a,c), LHX6 overexpression and knockout experiments^50,51^ (Extended Data Fig. 8d,e) and SOX6 overexpression experiments performed in cultured mouse primary neurons (Extended Data Fig. 9) each gave results congruent with SOX6 activation of both L1 expression and the PV interneuron transcriptional program.

To dissect L1 activation by SOX6, we generated an L1-mGreenLantern (L1-mGL) promoter reporter based on the 5′UTR of LRE3, another highly mobile human L1Hs element^52^. When this L1-mGL reporter was cotransfected into cultured HeLa cells with a SOX6-mCherry expression plasmid, 22% more GFP^+^ cells were observed on average than when cells were cotransfected with an mCherry control plasmid (Fig. 3b), a significant difference (*P* = 0.02). Inverting either LRE3 5′UTR SOX site, or scrambling the second site, did not reduce L1-mGL activity (Fig. 3a,b). Scrambling the first SOX site, however, reduced the percentage of GFP^+^ cells by 18% (*P* = 0.004), without SOX6 overexpression, and this reduction was greater (29%, *P* = 0.004) when cells were cotransfected with the SOX6-mCherry plasmid (Fig. 3b). In agreement with prior reports^11,12^, cotransfection with a SOX2 expression plasmid fully negated SOX6 activation of the L1-mGL reporter (Fig. 3c). Next, employing an L1-EGFP retrotransposition reporter^2,13,53^ to assay L1.3 (refs. ^41,42^) mobility in cultured HeLa cells, we found that stable SOX6 overexpression significantly (46%, *P* = 0.049) increased the percentage of GFP^+^ cells. Scrambling the first SOX site consistently reduced L1.3 retrotransposition efficiency by ~50%, in HeLa cells with and without stable SOX6 overexpression (Fig. 3d) and in cultured PA-1 embryonal carcinoma cells (Fig. 3e). These results demonstrated SOX6 activation of L1 promoter and retrotransposition reporters, dependent on the first L1Hs 5′UTR SOX site and attenuated by SOX2 expression.

### L1 promoter hypomethylation in PV interneurons

**Fig. 4:**
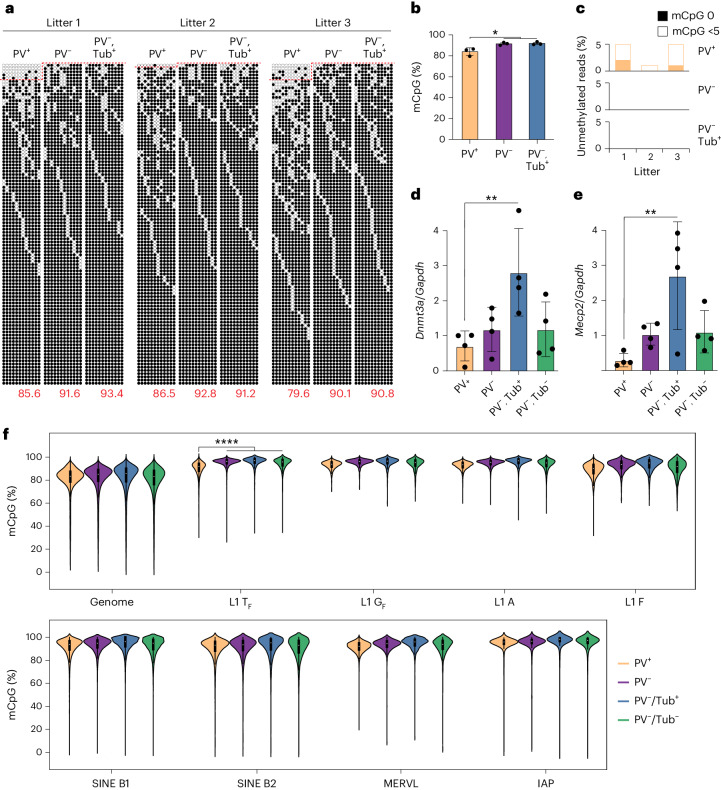
Global L1 T_F_ promoter hypomethylation in PV interneurons. **a**, Targeted bisulfite sequencing of L1 T_F_ promoter monomer CpG islands was performed on PV^+^, PV^−^ and PV^−^/Tub^+^ cells sorted from pooled hippocampal tissue from each of three neonate (P0) litters. Each cartoon displays 100 nonidentical randomly selected sequences, where methylated CpGs (mCpGs) and unmethylated CpGs are represented by black and white circles, respectively, as well as the overall mCpG percentage (red numbers). Amplicons above the dotted red line contain <5 mCpGs. **b**, L1 T_F_ monomer methylation was significantly lower (*P = 0.02, one-way ANOVA with Tukey’s multiple comparison test) in PV^+^ neurons than in either PV^−^ or PV^−^/Tub^+^ cells^.^ **c**, Fully (mCpG = 0) and nearly (mCpG <5) unmethylated L1 T_F_ monomers were found only in PV^+^ neurons. **d,** *Dnmt3a* mRNA abundance measured by qPCR in hippocampal cell populations, relative to *Gapdh*. **P = 0.01, one-way ANOVA with Dunnett’s multiple comparison test only to the PV^+^ population, *N* = 4 litters. **e**, As for **d**, except for *Mecp2*. **P = 0.005. **f**, CpG methylation ascertained by barcoded ONT sequencing upon matched hippocampal PV^+^ and PV^−^ cells from 11 separate neonate litter pools as well as PV^−^/Tub^+^ and PV^−^/Tub^−^ cells from one of those pools. Results are shown for the whole genome (divided into 10 kbp windows) and for CpG dinucleotides located within the 5′UTR of T_F_, G_F_, A-type and F-type L1s >6 kbp, or within B1 (>140 bp) and B2 (>185 bp) short interspersed elements (SINEs), and murine endogenous retrovirus L (MERVL) MT2 (>470 bp) and intracisternal A particle (IAP) (>320 bp) long terminal repeat sequences. Each included element accrued at least 20 methylation calls and 4 reads in each cell population. ****P = 0.0001, Kruskall–Wallis test comparing the PV^+^ population (*N* = 11 litter means) against the three PV^−^ populations combined ^(^*N* = 13 litter means) followed by Dunn’s multiple comparison test. Note: histogram data are represented as mean ± s.d. Source data

Despite apparent potential for SOX6-mediated L1 transcriptional activation, DNA methylation in somatic cells is expected to silence L1 promoters^17,25–30^. Therefore, to further probe the apparent specificity of L1 transcription to PV interneurons, we performed L1 T_F_ 5′UTR monomer bisulfite sequencing^25,46,54^ on neonate hippocampal cell populations. L1 T_F_ was significantly (*P* = 0.02) less methylated on average in PV^+^ neurons (83.9%) than in PV^−^ neurons (91.8%) (Fig. 4a,b). Unmethylated L1 T_F_ monomers were observed only in PV^+^ neurons (Fig. 4a,c). *Dnmt1*, *Dnmt3a* and *Mecp2* effect methylation-associated transcriptional repression in PV interneurons^30,55^. These genes all expressed markedly less mRNA in neonate PV^+^ neurons than in PV^−^ neurons (Fig. 4d,e and Supplementary Fig. 4a). MeCP2 protein expression was, on average, 10.5% lower in adult PV^+^ neurons compared to PV^−^ neurons (*P* = 0.0007) (Supplementary Fig. 4b,c). L1 repression thus appeared broadly relaxed in PV interneurons.

**Fig. 5:**
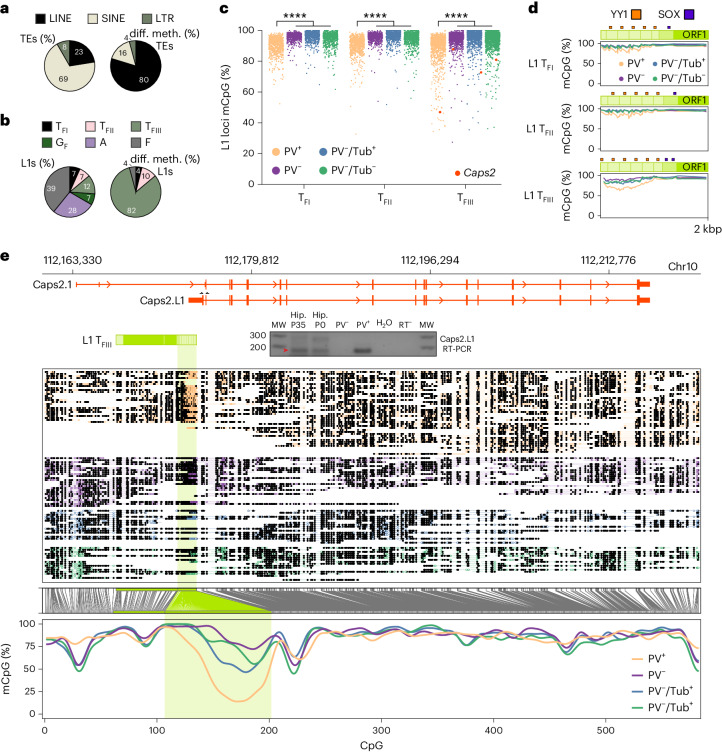
PV interneuron genes harbor hypomethylated L1 T_F_ promoters. **a**, Composition of all young (left) and differentially methylated (diff. meth.) (*P* < 0.01) young (right) retrotransposons, by superfamily. Note: MERVL (murine endogenous retrovirus L) and IAP (intracisternal A particle) are LTR retrotransposons. **b**, As per **a**, except showing the breakdown of young L1 subfamilies (left) and their contribution to the 50 differentially methylated loci showing the largest absolute change in methylation percentage. **c**, L1 T_FI_, T_FII_ and T_FIII_ subfamily CpG methylation strip plots for PV^+^, PV^−^, PV^−/^Tub^+^ and PV^-^/Tub^−^ cells, as represented collectively by the L1 T_F_ violin plot in Fig. 4f. Each point represents an L1 locus, with an example intronic to *Caps2* highlighted by an orange dot. ****P = 0.0001, Kruskall–Wallis test on PV^+^ population (*N* = 11 litter means) versus three PV^−^ populations combined (*N* = 13 litter means) followed by Dunn’s multiple comparison test. **d**, Composite DNA methylation profiles for the L1 T_F_ subfamilies displayed in **c**, representing the mean methylation for CpGs within the first 2 kbp of elements with six monomers. Darker green shading represents the nonmonomeric 5′UTR region. **e**, Methylation profile of the *Caps2* locus obtained from ONT sequencing. The first panel shows a full-length L1 T_FIII_ with intact ORFs, as highlighted in **c**, orientated antisense to the first intron of the canonical Caps2.1 isoform. ENCODE long-read transcriptome sequencing of hippocampus tissue (ENCLB505CBY) indicated a chimeric transcript, labeled here Caps2.L1, spliced into Caps2.1 exon 3 and encoding an ORF in frame with the Caps2.1 ORF. The gel image displays PCR products generated using primers specific to Caps2.L1 (marked by opposing black arrows), with input template cDNA from bulk adult (P35) hippocampus (5′RACE) and neonate (P0) hippocampus (reverse-transcribed total RNA from bulk and sorted PV^+^ and PV^−^ cells). The red arrow indicates on-target products confirmed by capillary sequencing. The second panel displays aligned ONT reads, with unmethylated CpGs colored in orange (PV^+^), purple ^(^PV^−^), blue (PV^−^/Tub^+^) and green (PV^−^/Tub^−^), and methylated CpGs colored black. The third panel indicates the relationship between CpG positions in genome space and CpG space, including those corresponding to the L1 T_FIII_ 5′UTR (shaded light green). The fourth panel indicates the fraction of methylated CpGs for each cell type across CpG space. MW, molecular weight. Source data

Long-read Oxford Nanopore Technologies (ONT) sequencing allows genome-wide analysis of retrotransposon family methylation, as well as that of individual retrotransposon loci^25,46,56^. We ONT sequenced PV^+^, PV^−^, PV^−^/Tub^+^ and PV^−^/Tub^−^ cells from neonatal hippocampus samples at ~16× average genome-wide depth, and ~29× and ~34× respectively for the PV^+^ and combined PV^−^ populations (Supplementary Table 1). Genome wide, DNA methylation was consistently, if subtly, lower in PV^+^ neurons than in PV^−^ cells (Fig. 4f). Among the potentially mobile retrotransposons surveyed, only the L1 T_F_ subfamily was significantly (*P* = 0.0001) less methylated in PV^+^ cells than in the PV^−^ populations (Fig. 4f). L1 loci supplied the vast majority (80%) of differentially methylated retrotransposons (Fig. 5a). Comparing PV^+^ and PV^−^ cells, all 638 differentially methylated (*P* < 0.01) full-length L1 T_F_ loci were also less methylated in the former population (Supplementary Table 2). Those genes containing at least one such L1 were significantly enriched (>20-fold, *P* < 0.01) for neurodifferentiation, regulation of GABAergic synaptic signaling, and cell–cell adhesion gene ontologies.

### Chimeric L1 transcripts can alter neuron complexity

**Extended Data Fig. 10:**
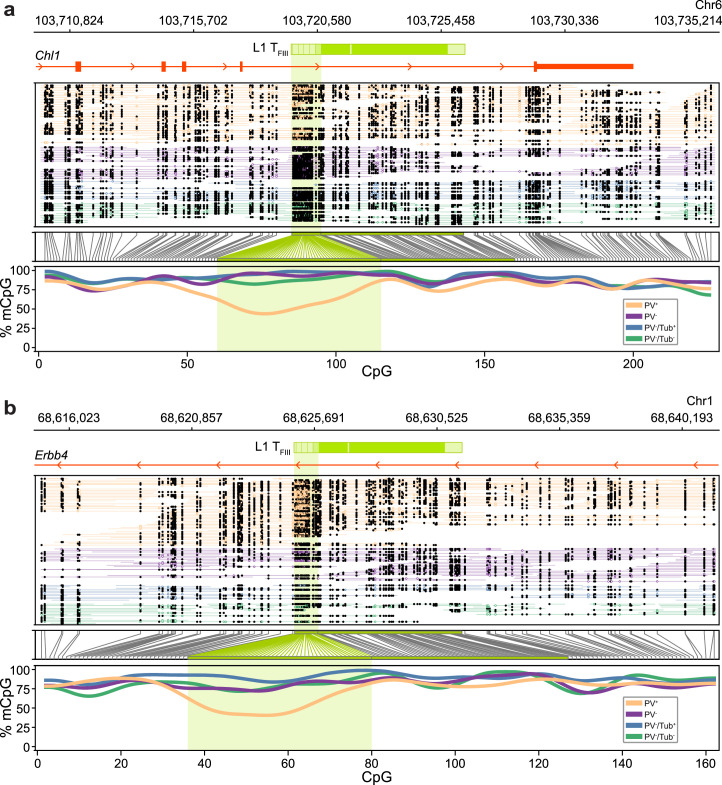
Hypomethylated L1s in PV interneuron genes, as identified by ONT sequencing. **a**, Methylation profile of a full-length L1 T_FIII_ element with intact ORFs, intronic to *Chl1*. The first panel shows the L1 orientated in sense to the last intron of *Chl1*. The second panel displays aligned ONT reads, with unmethylated CpGs colored in orange (PV^+^) and purple (PV^−^), blue (PV^−^/Tub^+^) and green (PV^−^/Tub^−^), and methylated CpGs colored black. The third panel indicates the relationship between CpG positions in genome space and CpG space, including those corresponding to the L1 T_FIII_ 5ʹUTR (shaded light green). The fourth panel indicates the fraction of methylated CpGs for each cell type across CpG space. **b**, As for (a), except displaying an L1 T_FIII_ antisense and intronic to *Erbb4*.

The T_F_ subfamily can be further divided into three subgroups, denoted T_FI_, T_FII_ and T_FIII_ (Fig. 5d), where T_FIII_ is the oldest and diverges in its 5′UTR when compared to T_FI/II_ (refs. ^57,58^). We found by far the highest fraction (82%) of strongly demethylated L1s corresponded to the T_FIII_ subfamily (Fig. 5b–d). Strikingly, we identified significantly (*P* < 0.01) hypomethylated L1 T_FIII_ copies with intact ORFs in the introns of genes expressed in PV interneurons, such as *Caps2* (calcyphosphine 2)^59^, *Chl1*, *Erbb4* and *Npsr1* (Fig. 5c,e, Extended Data Figs. 8b and 10 and Supplementary Table 2). In the case of *Caps2*, which expresses a calcium-binding protein in the same family as PV^59^, the L1 5′UTR was completely unmethylated in numerous PV^+^ neurons (Fig. 5e). Analysis of ENCODE PacBio long-read hippocampus transcriptome sequencing^60^ revealed a transcript initiated within and antisense^58^ to the L1 5′UTR and spliced downstream into a *Caps2* exon on the same strand. We termed this chimeric transcript Caps2.L1. By 5′RACE and reverse-transcription PCR (RT-PCR), we reliably detected Caps2.L1 in adult and neonate hippocampus tissue, and in PV^+^ cells, but not in PV^−^ cells (Fig. 5e). The predicted ORF for Caps2.L1 was in frame with that of the annotated canonical *Caps2* transcript, called here Caps2.1, and incorporated a novel N-terminal sequence (Fig. 5e).

**Fig. 6:**
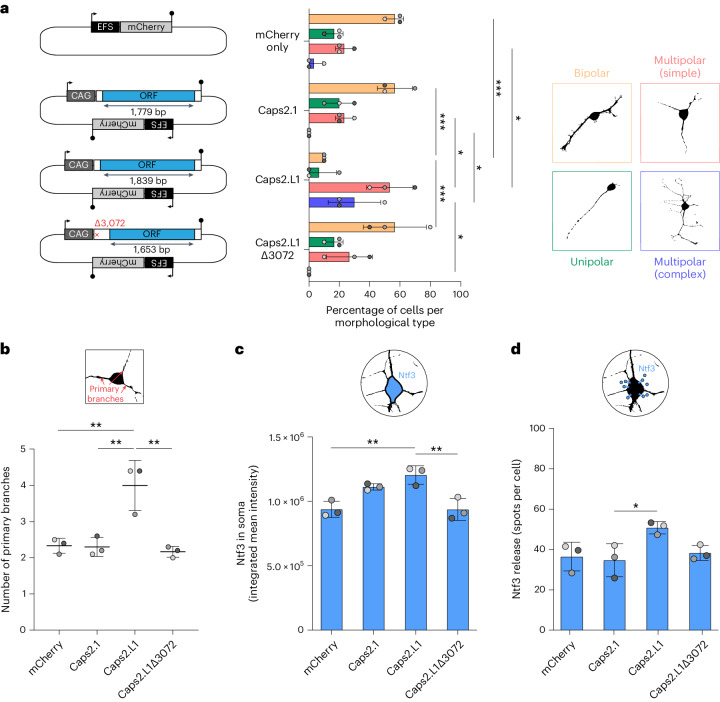
Caps2.L1 enhances neuron morphological complexity and Ntf3 release. **a**, *Caps2* mRNA expression construct design (left). Each is expressed from a CAG promoter. The Caps2.L1 ORF (blue) is larger than that of Caps2.1 and encodes an alternative N-terminus, whereas the Caps2.L1Δ3072 construct is identical to Caps2.L1 except with a 4 bp deletion at position 3,072 that results in a truncated Caps2.L1 ORF. All constructs have an mCherry cassette driven by an EFS promoter on their backbone. An empty mCherry vector was used as a transfection control. The black arrows represent promoters. The filled lollipops indicate polyadenylation signals. N2a neuroblastoma cells were transfected with each construct (left) and differentiated, and the morphology type (right) of mCherry^+^ cells was quantified (middle). Observed morphological types were classified as: (1) bipolar (orange), (2) unipolar (green), (3) multipolar (simple) (red) or (4) multipolar (complex) (blue). Each histogram data point represents the average of values from an independent experiment. *N* = 3 experiments, *n* = 10 cells per experiment. **b**, *Caps2* constructs assayed as in **a** but here showing the number of primary branches per cell. **c**, Ntf3 integrated mean immunofluorescence intensity in cell soma. *N* = 3 experiments, *n* = 95 cells per experiment. **d**, Ntf3 release quantified as Ntf3 spots per cell, outside of the soma. Analysis was performed on high-magnification confocal images within a fixed radius set around the cell soma. *N* = 3 experiments, *n* = 8–10 cells per experiment Note: in each histogram, experimental replicates are colored different shades of gray and data are represented as mean ± s.d. Significance values were calculated on the basis of the averages of independent experiments via two-way ANOVA with Šidák’s post-hoc test in **a** and one-way ANOVA with Tukey’s post-hoc test in **b**–**d**. *P < 0.05, **P < 0.01, ***P < 0.001. Source data

When introduced as an expression vector (Fig. 6a) into mouse N2a neuroblastoma cells, a tractable model of neurodifferentiation, Caps2.L1 significantly increased neuron branching and neurotrophin-3 (Ntf3) release, compared to Caps2.1 and controls (Fig. 6a–d). These results suggested that Caps2.L1 enhanced neuron morphological complexity and function. As the annotated *Caps2* promoter was fully methylated in PV^+^ neurons (Fig. 5e), we concluded Caps2.L1 is probably the canonical *Caps2* transcript isoform in mouse PV^+^ neurons, previously overlooked due to its initiation in the L1 T_FIII_ sequence. Finally, including the *Caps2* example, we identified 43 young mouse L1s whose 5′UTR promoted expression of a spliced transcript annotated by GenBank or detected by the abovementioned ENCODE PacBio transcriptome dataset (Supplementary Table 3). These results suggested unmethylated L1s can promote transcription of genes required for mouse PV interneuron development and function.

## Discussion

**Fig. 7:**
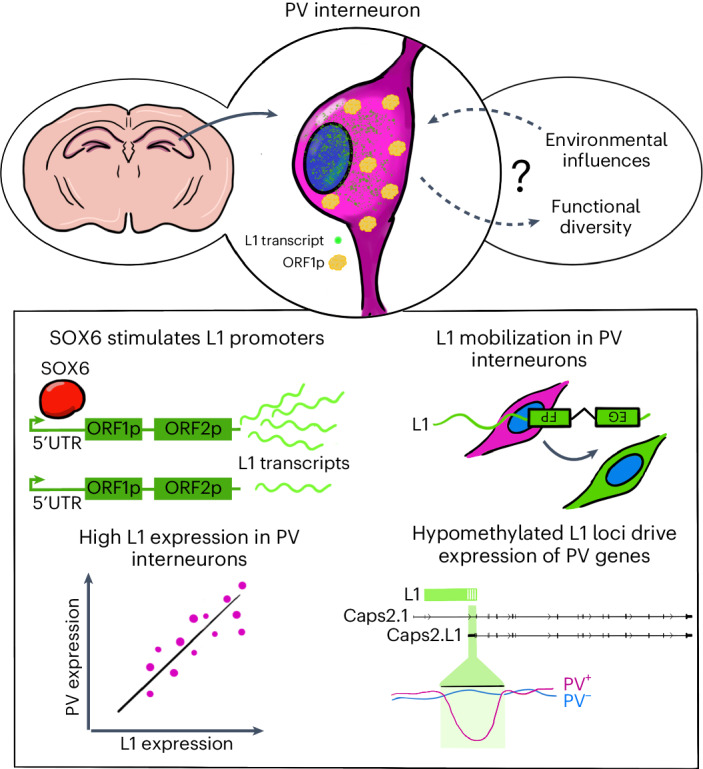
Model of L1 activation by SOX6 in mouse PV interneurons. Full-length L1 mRNA transcription and L1 protein expression occur in PV interneurons (top) and may be influenced by environmental cues or contribute to neuron functional diversity. L1 promoter elements are stimulated by SOX6 in the PV interneuron lineage (bottom), driving transcription of chimeric RNAs formed with adjacent genes, such as *Caps2*, and potentially increasing L1 mobility.

This study reveals L1 activity in the mouse PV interneuron lineage, most likely governed by SOX6 (Fig. 7). PV interneurons are ‘node’ cells that connect neural circuits associated with memory consolidation and other core cognitive processes^36,37^. The potential for L1 retrotransposition as a consequence of PV interneuron genes incorporating unmethylated mobile L1s is notable given the proposed roles for stochastic L1-mediated genome mosaicism in the brain^11^. Our results do not, however, preclude other neuronal lineages or brain regions from expressing L1 mRNAs and proteins, or supporting L1 retrotransposition. Engineered L1 reporter experiments have thus far generated data congruent with endogenous L1 mobility in the early embryo^7,8,10^, neurons^11,12,15–18^ and cancer^2,27^. While we and others have mapped endogenous L1 retrotransposition events in human^15–17^ and macaque^18^ neurons, the composition of the L1 T_F_ 3′UTR appears to severely impede such analyses in mouse^8,54^. A cL1_spa_ mouse model could in the future be used to evaluate mouse L1 mobilization in the PV interneuron lineage in vivo.

As highlighted here, retrotransposons can be integrated into transcriptional programs guiding cell fate^1,19–23^. Hypomethylated L1s could also influence the regulatory architecture of adjacent genes by attracting SOX proteins and their cofactors^33^. DNA methyltransferase activity appears to be moderately attenuated in PV interneurons. However, to explain L1 hypomethylation in this context, we favor a model where some L1 loci escape embryonic methylation and are transcribed^17,25^. Histone modifications associated with active transcription, perhaps enhanced by SOX6, then counteract their methylation in PV interneurons^61,62^. These heritable ‘escapee’ L1s, along with their transcriptional, regulatory and mobilization potential, are subject to evolutionary selection. Somatic retrotransposition could hence signify niches of L1 *cis*-regulatory innovation, as found here in the PV interneuron lineage.

## Methods

### Cultured cell L1 retrotransposition reporter assay

L1 retrotransposition efficiency was measured via an EGFP L1 reporter system in cultured HeLa and PA-1 cells, as described previously^13,17,53^. These assays employed the pCEP4_L1_eGFPI plasmid^17^ and tested the mobility of wild-type and RT mutant^2^ (D702A) L1.3 (refs. ^41,42^) sequences expressed from the native L1 promoter, as well as wild-type L1.3 sequences with their 5′UTR SOX binding sites scrambled or inverted^33^. Each SOX site mutation was introduced by fusion PCR to build an L1 fragment flanked by NotI (5′) and AgeI (3′) sites from two amplicons with overlapping primers that included the desired mutation. The complete amplicon was cloned within these sites in the original backbone, and its sequence was verified by capillary sequencing. In this L1 reporter^17^, the entire L1 3′UTR, with the thymine base deleted from within its native polyadenylation signal, preceded an EGFP reporter cassette activated only upon retrotransposition. EGFP expression was driven by a CBh (in HeLa) or cytomegalovirus (CMV) promoter (in PA-1). GFP^+^ cells were counted via flow cytometry. The L1 plasmid backbone incorporated a puromycin resistance gene. Three biological replicate assays were performed, each consisting of three wells per condition (technical replicates), on different days.

HeLa-JVM cells^2^ (obtained from the laboratory of John V. Moran) were cultured at 37 °C and 5% CO_2_ in HeLa complete medium (Dulbecco’s modified Eagle medium (DMEM); Life Technologies, cat. no. 11960044) supplemented with 10% fetal bovine serum (FBS; Life Technologies, cat. no. 10099141), 1% GlutaMAX (Life Technologies, cat. no. 35050061) and 1% penicillin–streptomycin (Life Technologies, cat. no. 15140122). Cells were passaged at 70–80% confluency using 0.25% trypsin–EDTA (Life Technologies, cat. no. 25200072). Briefly, 5 × 10^4^ HeLa cells were seeded per well of a six-well plate. Eighteen hours later, cells were transfected with 1 µg L1 plasmid per well using 3 µl FuGENE HD transfection reagent (Promega, cat. no. E2311) and 97 µl Opti-MEM (Life Technologies, cat. no. 31985047) per well according to the manufacturer’s protocol. Twenty-four hours post-transfection, the medium was replaced with HeLa complete medium. No puromycin selection was performed. The medium was replaced every other day, and cells were collected 6 days post-transfection by trypsinization, resuspended in sterile phosphate-buffered saline (PBS) and analyzed on a BD FACSymphony A5 SE Cell Analyzer (BD Biosciences) using FlowJo (version 10.8.1, BD Biosciences) to determine the percentage of GFP^+^ cells. Untransfected HeLa cells were used to set the GFP^−^ signal level in flow cytometry.

To assess L1 retrotransposition coincident with stable SOX6 overexpression, human SOX6 complementary DNA (NM_001145819.2) driven by a CBh promoter and mCherry driven by an elongation factor 1α short (EFS) promoter were inserted using BsrGI and Acc65I restriction enzymes (NEB, cat. nos. R3575S and R0599S, respectively) into the XLone-GFP plasmid (Addgene, cat. no. 96930). HeLa cells were cotransfected with this SOX6 expression plasmid and a hyperactive Piggybac transposase (HyPBase, a kind gift from the laboratory of Jose M. Polo) at a 3:1 ratio. The Piggybac plasmid harbored a blasticidin resistance gene, allowing transfected cells to be selected using 20 µg ml^−1^ of blasticidin for 2 weeks post-transfection to establish a stable SOX6-overexpressing HeLa cell line.

PA-1 cells were purchased from the American Type Culture Collection, cultured at 37 °C and 5% CO_2_, and maintained in Minimum Essential Medium (MEM) with GlutaMAX supplement (Life Technologies), 10% heat-inactivated FBS, 1× nonessential amino acids (Life Technologies) and 100 U ml^−1^ penicillin–streptomycin solution (Life Technologies). A total of 2 × 10^5^ PA-1 cells were seeded per well of a six-well plate and transfected as per the HeLa cell experiments above. The medium was replaced daily with PA-1 complete medium supplemented with 0.5 µg ml^−1^ puromycin the day after transfection, 1 µg ml^−1^ puromycin each day afterwards and 500 nM trichostatin A (Sigma, cat. no. T8552) 12 h before flow cytometry on day 6 after transfection. Flow cytometry employed a BD Accuri C6 Flow Cytometer (BD Biosciences). No untransfected PA-1 cells survived treatment with puromycin, ensuring that untransfected cells did not contribute to GFP^−^ cells on the day of analysis. Untransfected PA-1 cells not treated with puromycin were used to set the GFP^−^ signal level in flow cytometry. As a quality check, plasmid transfection efficiencies were calculated by cotransfecting with pCEP-EGFP.

### cL1_spa_ retrotransposition in primary neuronal cultures

We designed a Cre-LoxP conditional L1 retrotransposition reporter, cL1_spa_, based on a variant of the mobile L1 T_F_ element L1_spa_ (refs. ^44,63^). Here, L1_spa_ was contained in a pCEP4 backbone, with ORF1p corrected at two amino acid positions to match the L1 T_F_ subfamily consensus sequence, and had an mCherry indicator cassette driven by an EF-1ɑ promoter embedded in its 3′UTR^45,46^. The G-rich region of the 3′UTR (nucleotides 7,260–7,416) was moved downstream of the reporter cassette, cloned between two BamHI sites before the SV40 polyadenylation signal^45^. To prohibit full-length L1_spa_ mRNA production in the absence of Cre recombinase, between the L1_spa_ 5′UTR promoter and ORF1 we inserted a LSL cassette containing two LoxP sequences (ATAACTTCGTATAGCATACATTATACGAAGTTAT) flanking a 786 bp sequence containing three SV40 polyadenylation signals and followed by a Kozak consensus sequence (Vector Builder). We synthesized a fragment containing the 5′UTR, LoxP-Stop-LoxP and ORF1 (GenScript) sequences, flanked by Not1 and PacI restriction sites, and cloned it into the L1_spa_ backbone. We used this construct to electroporate primary neuronal cells using a Neon NxT Electroporation System (Thermo Fisher Scientific) and the Neon Transfection System 10 µl Kit (Thermo Fisher Scientific, cat. no. MPK1025,) at 1,500 V, 10 ms and three pulses. A total of 2.5 µg of plasmid was used per electroporation, with two electroporations plated per 12-well plate well.

Primary neuronal cultures were prepared from embryonic day (E)18 mice by dissecting the cortices and placing them in a papain solution (Worthington Biochemical, cat. no. LS003126) dissolved in a basic culture medium (Neurobasal medium (Gibco, cat. no. 21103-049), GlutaMAX (Gibco, cat. no. 35050061) and penicillin–streptomycin (5,000 U ml^−1^; Gibco, cat. no. 15070063)). Cortices were incubated for 20 min at 37 °C with pipette mixing every 5 min. Cells were then filtered through a 70 µm cell strainer into an inactivation solution containing albumin-ovomucoid inhibitor (Worthington, cat. no. LK003182), DNAseI (1 mg ml^−1^; Sigma, cat. no. DN-25) and basic culture medium with 5% FBS. Cells were centrifuged at 400*g* for 2 min at room temperature and then resuspended in 5% FBS culture medium and counted. A total of 250,000 cells were washed with PBS twice and used for one electroporation. Cells were then plated on coverslips coated with poly-d-lysine (1 mg ml^−1^; Sigma, P64407). Four days post-electroporation, cells were transduced with a lentiviral construct expressing Cre-T2A-TagBFP driven by an EFS moderate EF-1α core promoter (Vector Builder). Four days post-transduction, cells were processed for either immunostaining or DNA extraction. For immunostaining, culture medium was removed, and cells were immediately fixed in 4% paraformaldehyde (PFA) (20 min, 4 °C) and then washed with PBS. Cells were permeabilized for 5 min with PBT (PBS with 0.5% Triton) and then incubated with blocking buffer (10% normal donkey serum (Jackson ImmunoResearch, cat. no. AB2337258) in PBT) for 1 h at room temperature. Cells were incubated overnight with the following primary antibodies: goat anti-tdTomato (Sicgen, cat. no. AB8181, 1:2,000), mouse anti-Tub (Sigma, cat. no. T4026, 1:1,000) and either rabbit anti-Cre (Cell Signaling, cat. no. 15036, 1:500) or rabbit anti-PV (Swant, PV27, 1:500). Cells were washed with PBS for 15 min, three times at room temperature and then incubated with secondary antibodies: Cy3 donkey anti-goat (Jackson ImmunoResearch, cat. no. 715-165-150, 1:500), Alexa Fluor 488 donkey anti-mouse (Jackson ImmunoResearch, cat. no. 715-546-150, 1:1,000) and Alexa Fluor 647 donkey anti-rabbit (Jackson ImmunoResearch, cat. no. 711-606-152, 1:1,000). Hoechst 33258 (Sigma, cat. no. B2883) was added to the secondary antibody mix to stain nuclei. Cells were washed with PBS for 15 min, three times at room temperature, then air dried and mounted using an aqueous fluorescence mounting medium (Agilent Dako, cat. no. S302380-2). Cells were visualized and imaged using an Olympus UPLXAPO 20×/0.8 numerical aperture (NA) air objective on a spinning-disk confocal microscope (SpinSR10; Olympus) built around an Olympus IX3 body and equipped with two ORCA-Fusion BT sCMOS cameras (Hamamatsu Photonics K.K.) and controlled by Olympus cellSens software.

Genomic DNA was phenol–chloroform extracted. Oligonucleotide PCR primers spanning the mCherry cassette exon–exon junction (Supplementary Table 4) were purchased from Integrated DNA Technologies. PCR reactions were prepared using the MyTaq DNA polymerase kit (Bioline, cat. no. BIO-21111). Reactions contained a 5× MyTaq Buffer, 10 pmol of each primer, 1 µl DNA input template (20 ng) and 0.2 µl Taq polymerase in 25 μl final volume. Cycling conditions were as follows: 95 °C for 2 min, followed by 30 cycles of 95 °C, 15 s; 58 °C, 15 s; 72 °C, 50 s and extension 72 °C, 5 min. PCR products were separated on 1.5% agarose gel.

### Cultured cell L1 promoter reporter assay

The effects of SOX site mutations on L1 promoter activity were assessed in cultured HeLa-JVM cells using the LRE3 (ref. ^52^) 5′UTR to drive expression of a mGreenLantern^64^ (mGL) green fluorescent protein. The native LRE3 5′UTR and SOX site mutants were ordered as synthetic double-stranded DNA gene blocks from Integrated DNA Technologies and incorporated EcoRI and BamHI restriction sites at their 5′ and 3′ ends, respectively. These sites were used to clone the LRE3 5′UTR into an expression vector, resulting in an L1 promoter reporter. SOX protein overexpression was evaluated using the L1 promoter reporter in HeLa cells in two experiments. In the first experiment, cells were seeded at 1 × 10^5^ cells per well in a six-well plate. Sixteen hours after seeding, cells were cotransfected with 3 µl FuGENE HD, 97 µl Opti-MEM and a 1:1 ratio of the L1 promoter reporter and a plasmid encoding the human SOX6 (NM_001145819.2) cDNA, driven by a CBh promoter and including an mCherry cDNA under the control of an EFS promoter to assess transfection efficiency (Vector Builder). This plasmid, without SOX6, was used as a control. The transfection medium was replaced after 24 h with DMEM-complete medium and the cells were incubated for 24 h, after which the percentage of GFP^+^ cells was determined by flow cytometry. In the second experiment, WT and stably SOX6-overexpressing cells were seeded at 1 × 10^5^ cells per well in a six-well plate. Sixeen hours after seeding, both cell lines were cotransfected with 3 µl FuGENE HD, 97 µl Opti-MEM and a 1:1 ratio of the L1 promoter reporter and a plasmid encoding SOX2 cDNA (NM_003106.4) under a CBh promoter (Vector Builder). This plasmid also included an EFS promoter driving the expression of mCherry to monitor transfection efficiency. The same plasmid without SOX2 was used as a control. Twenty-four hours later, the transfection medium was replaced with DMEM-complete medium and cells were incubated for 48 h, after which the percentage of GFP^+^ cells was analyzed by flow cytometry (BD FACSymphony A5 SE Cell Analyzer, BD Biosciences) as above.

### SOX6 expression assay in primary neuronal cultures

Primary neuronal cultures were transduced at day 5 post plating with 1 µl per well of 12-well plate using either adeno-associated virus of serotype 2 (AAV2) encoding the mouse SOX6 (NM_011445.4) cDNA or mCherry control at a concentration of >2 × 10^11^ genome copies (GC) per milliliter. SOX6 expression was driven by a CBh promoter, and the construct included an mCherry cassette under the control of an EFS promoter to assess transduction efficiency (Vector Builder). Twenty-four hours after transduction, RNA was extracted using TRIzol reagent (Invitrogen, cat. no. 15596026) following the manufacturer’s protocol. SYBR Green qPCR was prepared using the Power SYBR Green RNA-to-CT 1 step kit (Applied Biosystems, cat. no. 4391112) and specific SOX6 primers (mSOX6_F and mSOX6_R) to assess SOX6 overexpression. Reactions contained a 2× Power SYBR Green RT-PCR Mix, 10 pmol of each primer, 1 µl RNA input template and 1× RT enzyme mix in a 10 μl final volume. Cycling conditions were as follows: 48 °C for 30 min, 95 °C for 10 min, followed by 40 cycles of 95 °C, 15 s; 60 °C, 1 min. To assess potential DNA contamination, an L1 T_F_ qPCR using primers L1Md_5UTR_F and L1Md_5UTR_R was performed with and without RT. A three or more cycle difference between experiments run with and without RT, and detection after cycle 30 in the latter, was considered as non-DNA-contaminated RNA. A TaqMan assay was also performed using Applied Biosystems custom L1 and 5S ribosomal RNA (rRNA) TaqMan MGB probes, as listed in Supplementary Table 4. TaqMan qPCR reactions contained: 4× TaqPath 1-Step RT-qPCR multiplex reaction master mix (ThermoFisher, cat. no. A28521), 4 pmol of each primer, 1 pmol probe and 1 µl RNA (100–150 ng) input template in a 10 µl final volume. Cycling conditions were as follows: 37 °C for 2 min; 50 °C for 15 min; 95 °C for 2 min, followed by 40 cycles of 95 °C, 3 s; 60 °C, 30 s. TaqMan assays for L1 probe conjugated with VIC (2’-chloro-7’-phenyl-1,4-dichloro-6-carboxyfluorescein) were multiplexed with an assay for 5S rRNA control, conjugated to 6FAM (6-Carboxyfluorescein) fluorophores. Primer/probe sequences and the associated detection channels are listed in Supplementary Table 4. Seventy-two hours after transduction, the culture medium was removed, and cells were fixed in 4% PFA (20 min, 4 °C) and washed with PBS. Immunohistochemistry was then performed as for mouse primary neuronal cultures. For ORF1p immunostaining analysis, *Z*-stack images were acquired using the SoRa super-resolution disk and 3.2× magnification. Image processing and analysis post-acquisition for the morphology analysis were performed using Fiji for Windows (ImageJ 1.52d). ORF1p intensity analysis in mCherry^+^ cells was performed using Imaris 9.5.1 (Bitplane, Oxford Instruments). Integrated mean intensity was calculated as equal to area times mean intensity value and normalized to the mCherry control for the respective experiment.

### L1-EGFP transgenic mice

To trace retrotransposition of an engineered L1 reporter in vivo, we generated a new transgenic L1-EGFP mouse line harboring L1.3, with epitope tags on ORF1p and ORF2p and an EGFP indicator cassette^2,13^ embedded in its 3′UTR. To assemble the L1 transgene, we cloned the NotI-BstZ17I fragment from pJM101/L1.3-ORF1-T7-ORF2-3×FLAG (containing T7 gene 10 epitope tag on the C-terminus of ORF1 and a 3×FLAG tag on the C-terminus of ORF2) into p99-GFP-LRE3, yielding p99-GFP-L1.3-ORF1-T7-ORF2-3×FLAG. In p99-GFP-L1.3-ORF1-T7-ORF2-3×FLAG, transgene transcription was driven by the native L1.3 promoter, with an SV40 polyadenylation signal (pA) located downstream of the EGFP retrotransposition indicator cassette. The EGFP cassette was equipped with a CMV promoter and a herpes simplex virus type 1 (HSV) thymidine kinase (TK) polyadenylation signal, facilitating EGFP expression upon genomic integration via retrotransposition. In preparation for pronuclear injection, EGFP-L1.3-ORF1-T7-ORF2-3×FLAG was released by digestion with Not1 and MluI restriction enzymes, separated from the vector backbone on a 0.7% agarose gel, purified by phenol–chloroform extraction and eluted in microinjection buffer (7.5 mM Tris–HCl and 0.15 mM EDTA pH 7.4). Transgenic L1-EGFP mice were produced by the Transgenic Animal Service of Queensland, University of Queensland, using a standard pronuclear injection protocol. Briefly, zygotes were collected from superovulated C57BL/6 females. The microinjection buffer containing EGFP-L1.3-ORF1-T7-ORF2-3×FLAG was then transferred to the zygote pronuclei. Successfully injected zygotes were transplanted into the oviducts of pseudopregnant females. Primers flanking the EGFP cassette were used to screen potential founders by PCR (Supplementary Table 4). Identified founder L1-EGFP animals were bred on a C57BL/6 background. All procedures were followed as approved by the University of Queensland Animal Ethics Committee (TRI/UQ-MRI/381/14/NHMRC/DFG and MRI-UQ/QBI/415/17).

### Histology

Adult C57BL/6 and transgenic L1-EGFP mice (12 weeks) were anesthetized using isoflurane and perfused intracardially with PBS and 4% PFA. CD1 pups, having been electroporated in utero with mouse L1-EGFP plasmids, were killed at postnatal day 10 by cervical dislocation. CBA×C57BL/6 mice (12 weeks), intended for RNA FISH, were injected intraperitoneally with sodium pentobarbital (50 mg kg^−1^), followed by cervical dislocation to ensure killing. All brains were dissected and fixed in PFA for 24 h. For cryopreservation, fixed brains were immersed first in 15% sucrose and then 30% sucrose to submersion, and embedded in optimal cutting temperature compound and stored at −80 °C. C57BL/6 and transgenic L1-EGFP brains were sectioned on a cryostat (Leica, settings object temperature (OT) −20 °C, chamber temperature (CT) −20 °C) at 40 µm thickness. Free-floating sections were collected in PBS and stored at 4 °C. CBA×C57BL/6 brains were sectioned on a cryostat (Leica, settings OT −22 °C, CT −22 °C) at 30 µm thickness. Free-floating sections were collected in cryoprotectant (25% glycerol and 35% ethylene glycol, in PBS) and immediately stored at −20 °C.

Tissue processing and immunofluorescent staining with primary and secondary antibodies were carried out as described previously^65^. Primary antibodies and dilutions were as follows: rabbit anti-GFP, 1:500 (Thermo Fisher A11122); chicken anti-GFP, 1:500 (Millipore AB16901); mouse anti-T7, 1:500 (Millipore, cat. no. 69522); rabbit anti-T7, 1:500 (Millipore, AB3790); goat anti-tdTomato, 1:1,000 (Sicgen, cat. no. AB8181); mouse anti-NeuN, 1:250 (Millipore, cat. no. MAB377); guinea pig anti-NeuN, 1:250 (Millipore, cat. no. ABN90), rabbit anti-Gad65/67 (Gad1), 1:500 (Sigma, cat. no. G5163); mouse anti-PV, 1:2,000 (Sigma, cat. no. P3088); rabbit anti-β tubulin III (Tub), 1:500 (Sigma, cat. no. T2200); rabbit anti-MeCP2, 1:500 (Abcam, cat. no. ab2828), rabbit anti-ORF1p (Abcam, cat. no. ab216324). Secondary antibodies and dilutions were as follows: donkey anti-guinea pig Dylight 405, 1:200 (Jackson Immunoresearch, cat. no. 706475148); donkey anti-mouse Dylight 405, 1:200 (Jackson Immunoresearch, cat. no. 715475150); donkey anti-chicken Alexa Fluor 488, 1:500 (Jackson Immunoresearch, cat. no. 703546155); donkey anti-rat Alexa Fluor 488, 1:500 (Jackson Immunoresearch, cat. no. 712546150); donkey anti-rabbit Alexa Fluor 488, 1:500 (Thermo Fisher, cat. no. A21206); donkey anti-goat Alexa Fluor 594, 1:500 (Jackson Immunoresearch, cat. no. 705586147); donkey anti-rabbit Cy3, 1:200 (Jackson Immunoresearch, cat. no. 711165152); donkey anti-mouse Cy3, 1:500 (Jackson Immunoresearch, cat. no. 715165150); donkey anti-guinea pig Alexa Fluor 647, 1:500 (Millipore, cat. no. AP193SA6); donkey anti-mouse Alexa Fluor, 1:500 (Jackson Immunoresearch, cat. no. 715606150). For nuclei labeling, BisBenzimide H33258 (Sigma, cat. no. B2883) was used. For blocking serum, normal donkey serum (Jackson Immunoresearch, cat. no. 017000121) was used.

### Imaging of brain sections

EGFP^+^ cells were imaged on a Zeiss LSM510 confocal microscope. Acquisition of high magnification, *Z*-stack images was performed with Zen 2009 software. Images of EGFP, NeuN and PV immunostaining for quantification were taken from hippocampal and adjacent cortical areas using a Zeiss AxioObserver Z1 microscope and Zen 2009 software, equipped with an ApoTome system and a 10× objective. Visualization and imaging of EGFP, NeuN and PV in in utero electroporated mice was performed using a Zeiss Plan-Apochromat 20×/0.8 NA air objective and a Plan-Apochromat 40×/1.4 NA oil-immersion objective on a confocal/two-photon laser-scanning microscope (LSM 710, Carl Zeiss Australia) built around an Axio Observer Z1 body and equipped with two internal gallium arsenide phosphide (GaAsP) photomultiplier tubes (PMTs) and three normal PMTs for epi- (descanned) detection and two external GaAsP PMTs for nondescanned detection in two-photon imaging, and controlled by Zeiss Zen Black software.

RNA FISH for sections of hippocampus and adjacent cortical areas, as well as MeCP2, NeuN and PV immunostainings were imaged on a spinning-disk confocal system (Marianas; 3I, Inc.) consisting of a Axio Observer Z1 (Carl Zeiss) equipped with a CSU-W1 spinning-disk head (Yokogawa Corporation of America), ORCA-Flash4.0 v2 sCMOS camera (Hamamatsu Photonics), using a 63×/1.4 NA C-Apo objective and a 20×/0.8 NA Plan-Apochromat objective, respectively. All *Z*-stack spinning-disk confocal image acquisition was performed using SlideBook 6.0 (3I, Inc.).

PV stereology was performed on an upright Axio Imager Z2 fluorescent microscope (Carl Zeiss) equipped with a motorized stage and Stereo Investigator software (MBF Bioscience). Contours were drawn on the basis of 4′,6-diamidino-2-phenylindole (DAPI) staining using a 5×/0.16 NA objective. Counting was performed on a 10×/0.3 NA objective. All image processing and analysis post acquisition was performed using Fiji for Windows (ImageJ 1.52d). ORF1p was imaged using an Olympus UPLXAPO 20×/0.8 NA air objective on a spinning-disk confocal microscope (SpinSR10; Olympus).

### Single-molecule RNA FISH

Two custom RNAscope probes were designed against the RepBase L1 T_FI_ subfamily consensus sequence (Extended Data Fig. 4). L1 probe A (design #NPR-0003768, Advanced Cell Diagnostics, cat. no. ADV827911C3) targeted the L1 T_FI_ 5′UTR monomeric and nonmonomeric region (consensus positions 827–1,688). L1 probe B (design #NPR-000412, Advanced Cell Diagnostics, cat. no. ADV831481C3) targeted the L1 T_FI_ 5′UTR monomeric region (consensus positions 142–1,423). Weak possible off-target loci for probe A and B comprised the pseudogene Gm-17177, two noncoding RNAs (LOC115486508 for probe A and LOC115490394 for probe B) and a minor isoform of the *Ppcdc* gene (only for probe A), none of which was expressed beyond very low levels or with specificity to PV^+^ neurons. Using the L1 T_F_ RNAscope probes, we performed FISH on fixed, frozen brain tissue according to the manufacturer’s specifications (RNAscope Fluorescent Multiplex Reagent Kit part 2, Advanced Cell Diagnostics, cat. no. 320850) and with the following modifications: 30 μm coronal sections instead of 15 μm, and boiling in target retrieval solution for 10 min instead of 5 min. To identify neurons, we performed immunohistochemistry using a rabbit anti-Tub antibody (Sigma, cat. no. T2200) and donkey anti-rabbit Cy3 secondary antibody (Jackson Immunoresearch, cat. no. 711165152) following a previously described protocol^65^. To identify PV^+^ neurons we employed a validated mouse PV RNAscope probe (Mm-Pvalb-C2, Advanced Cell Diagnostics, cat. no. ADV421931C2). Probes for the ubiquitously expressed mouse peptidylprolyl isomerase B (*Ppib*) gene and *Escherichia coli* gene *dapB* were used as positive and negative controls, respectively, for each FISH experiment.

### Cell quantifications

We analyzed four hippocampal sections per animal for each L1 T_F_ 5′UTR probe (Extended Data Fig. 4) using Imaris 9.5.1 (Bitplane, Oxford Instruments). To render three-dimensional (3D) visualizations for a given neuron, we used Tub and DAPI staining to outline its soma and nucleus along *Z*-stack planes where the cell was detected. We set voxels outside the cell, and inside the nucleus, to a channel intensity value of zero to only retain cytoplasmic L1 mRNA signal and avoid nuclear L1 DNA. We then used the Imaris Spots module to detect L1 mRNA puncta and calculated the number of L1 spots within the cytoplasm in PV^+^/Tub^+^ versus PV^−^/Tub^+^ neurons.

We quantified MeCP2 protein expression using Imaris 9.5.1 (Bitplane, Oxford Instruments), and we analyzed two hippocampal sections per animal. For each cell, we drew the contours of NeuN immunostaining along the relevant *Z*-stack planes and rendered a cell 3D visualization. We then calculated the mean MeCP2 channel intensity in PV^+^/NeuN^+^ and PV^−^/NeuN^+^ neurons. For PV stereology, we stained and analyzed every 12th hippocampal section per animal. Cell density was calculated using the total number of PV^+^ cells and the total subregion area from approximately six sections per animal.

To quantify EGFP^+^ cells we stained and analyzed every 12th hippocampal section (again, ~6 sections per animal). To visualize colocalization, we used Adobe Photoshop CC 2017. We counted EGFP^+^, EGFP^+^/NeuN^+^ and EGFP^+^/PV^+^ cells across the hippocampus and adjacent cortex. The average number of double-labeled cells per 100 mm^2^ was determined for each animal. All statistical analyses were performed using Prism (v9.5.1).

To quantify L1 ORF1p expression, we analyzed two hippocampal sections per animal. We used Imaris 9.5.1 (Bitplane, Oxford Instruments) Surface function to create DG and CA regions of interest and mask selection function to apply the surface to NeuN, ORF1p and PV channels. We then used the spots function (automated cell detection for minimum diameter of 13 µm) to identify and quantify NeuN^+^ neurons in each area. We calculated the mean ORF1p and PV channel intensities within each NeuN^+^ neuron. We defined ORF1p^+^ and PV^+^ neurons as those with a mean intensity above a background spot mean intensity.

### L1 T_F_ 5′RACE

Total RNA was extracted from pooled C57BL/6 bulk adult (12 weeks) hippocampus tissue and sorted PV^+^ neurons obtained from pooled neonate (postnatal day 1 or 2) hippocampus tissue using TRIzol. 5′RACE was performed using the 5′RACE module of the Takara SMARTer 5′/3′ RACE Kit (cat. no. 634858) according to the manufacturer’s protocol. Ten nanograms of total RNA was used as input for each reaction, and two reactions from independent experiments were performed. 5′RACE cDNA was PCR amplified using an L1-specific primer (Supplementary Table 4) and the 5′RACE universal primer provided in the Takara kit. Thirty-five PCR cycles were performed as follows: 94 °C for 30 s, 67 °C for 30 s, 72 °C for 3 min. PCR products were visualized on a 1% agarose gel. The resultant smear was excised, and PCR fragments were purified using conventional phenol–chloroform DNA extraction. Iso-Seq template preparation using the Iso-Seq Express kit followed by PacBio SMRT Cell sequencing on a PacBio Sequel II platform was performed by the Australian Genome Research Facility. Four samples (two replicates each of bulk and PV^+^ hippocampus cells) were multiplexed on a single SMRT cell. Reads were aligned to the mouse reference genome (mm10) using minimap2 version 2.20 (ref. ^66^) (parameters -t 96 -N 1000 -p 0.95 -ax splice:hq -uf) and sorted with samtools version 1.12 (ref. ^67^). Uniquely mapped reads, that is, those that aligned to one genomic position only at their best alignment score and as a primary alignment, were retained if an L1-specific primer and a 5ʹRACE universal primer were located at each of their termini. Reads were then assigned to the full-length L1 T_FI_ elements they overlapped, with alignments required to terminate within the L1 body. The start positions of these alignments within the 5′UTR or upstream of the L1 were then recorded as putative TSSs supported by at least one read. Replicates gave very similar results and were pooled for display purposes.

### Nanopore methylation analysis

Genomic DNA was phenol–chloroform extracted from PV^+^ and PV^−^ (Supplementary Fig. 1) populations purified from ten neonate (P0) littermate hippocampus sample pools. Additional DNA was obtained from the PV^+^, PV^−^, PV^−^/Tub^+^ and PV^−^/Tub^−^ populations of another neonate pool. DNA samples were sheared to ~10 kb average size, prepared as barcoded libraries using a Ligation Sequencing Kit (ONT, SQK-LSK109), and sequenced on an ONT PromethION platform (Garvan Sequencing Platform). Bases were called in SLOW5 format^68^ with Buttery-eel^69^ using Guppy 4.0.11 (ONT) and reads aligned to the mm10 reference genome using minimap2 version 2.20 (ref. ^66^) and samtools version 1.12 (ref. ^67^). Reads were indexed and per-CpG methylation calls generated using nanopolish version 0.13.2 (ref. ^70^). Methylation likelihood data were sorted by position and indexed using tabix version 1.12 (ref. ^71^). Methylation statistics for the genome divided into 10 kbp bins, and reference retrotransposons defined by RepeatMasker coordinates (http://www.repeatmasker.org/), were generated using MethylArtist version 1.0.4 (ref. ^72^), using commands db-nanopolish, segmeth (parameters:–excl_ambig and–primary_only) and segplot with default parameters. Only full-length (>6 kbp) L1s were included, with methylation measured on 5′UTR CpGs. Methylation profiles for individual loci were generated using the MethylArtist command locus, with a 28 bp sliding window, and composite profiles with the MethylArtist command composite, with parameters used elsewhere^25^. To identify individual differentially methylated retrotransposons (Supplementary Table 2), we required elements to have at least four reads and 20 methylation calls in each sample being compared. Comparisons were carried out via Fisher’s exact test using methylated and nonmethylated call counts, with significance defined as a Bonferroni-corrected *P* value of less than 0.01. Gene Ontology enrichment analysis was performed on genes containing at least one differentially methylated L1 T_F_ sequence with PANTHER, using Fisher’s exact test and Bonferroni correction.

### Caps2.L1 expression assays in N2a cells

Differentiating mouse N2a (neuro-2a) neuroblastoma cells (American Type Culture Collection, cat. no. CCL-131) were used to assess the impact of Caps2.L1 expression on neuronal phenotype. To generate a Caps2.L1 expression plasmid, we GenScript gene synthesized the Caps2.L1 chimeric transcript, as detected by ENCODE long-read PacBio sequencing of bulk hippocampus tissue (ENCLB505CBY) and cloned it to be under the control of a CAG promoter (Vector Builder). To create a same-size control plasmid, we destroyed the *KpnI* restriction site in Caps2.L1, resulting in *Δ3072* truncation of the Caps2.L1 ORF. As an additional control, we generated a plasmid expressing the annotated canonical Caps2.1 transcript (RefSeq: NM_178278.4), also driven by a CAG promoter (Vector Builder). Each plasmid included an EFS promoter driving mCherry to identify cells receiving the constructs. An empty plasmid, containing only EFS driving mCherry expression was used as an additional control. Cells were cultured at 37 °C in 5% CO_2_ in high-glucose (4.5 g l^−1^) DMEM, supplemented with 10% FBS and 100 U ml^−1^ penicillin–streptomycin solution (Life Technologies). A total of 1.2 × 10^5^ cells per well were plated on coverslips coated with 0.1% gelatine in 12-well plates and transfected with 500 ng of plasmid DNA per well using Lipofectamine 2000 (Invitrogen, cat. no. 11668019) following the manufacturer’s instructions. Twenty-four hours post-transfection, the medium was replaced with a differentiation medium containing high-glucose DMEM with 0.5% FBS and 5 µM retinoic acid.

After 5 days of differentiation, the culture medium was removed, and cells were fixed in 4% PFA (20 min, 4 °C) and washed with PBS. Immunohistochemistry was then performed as for mouse primary neuronal cultures. For morphology analysis, *Z*-stack images were acquired using the SoRa super-resolution disk and 3.2× magnification. Image processing and analysis post-acquisition for the morphology analysis were performed using Fiji for Windows (ImageJ 1.52d). Ntf3 analysis was performed using Imaris 9.5.1 (Bitplane, Oxford Instruments). To quantify Ntf3 release, circular outlines (30 µm diameter) were manually added to 3D visualizations of neuronal soma (for mCherry-positive neurons) along the *Z*-stack planes where the cell was detected. Voxels outside the outline were set to a channel intensity value of zero to retain only the Ntf3 signal of a given cell. We then manually placed and quantified the number of spots seen as Ntf3 signal dots outside cell soma. For Ntf3 analysis inside the soma, we used the automatic Imaris surface function to render the surface of mCherry-positive cells and exported mean intensities and area. Integrated mean intensity was calculated as equal to area times mean pixel value.

### Statistics and reproducibility

Statistical methods, error bars (standard deviation, s.d.), *P* values and replicate or sample sizes (*n* or *N*) are indicated in figure legends. Statistical tests were implemented with GraphPad Prism (version 9.5.1) with the exception of Supplementary Tables 2 and 3, where Fisher’s exact test was implemented with SciPy (version 1.4.1). Data distributions were assumed to be normal, but this was not formally tested and individual data points are displayed where relevant on graphs. No statistical method was used to predetermine sample size. Experiments were designed to obtain at least biological triplicate data. No data were excluded from the analysis. Stratified randomization was used on the basis of age to allocate mice to experimental groups. Cell culture experiments were not randomized. Blinding to group allocation was used for image analyses involving different treatment groups. No blinding to sample identity during sample collection was used. Immunostainings of brain sections (Figs. 1b–e and 2f, Extended Data Figs. 1b–f and 3a–c and Supplementary Fig. 4c), cell culture assays (Fig. 1k–m and Extended Data Figs. 2b–d, 4f,g, 7 and 9a,d,e) and RNAscope experiments (Fig. 2a and Extended Data Fig. 4c–e) were repeated independently at least twice. L1 promoter and retrotransposition assays (Fig. 3b–e) as well as the Caps2 expression plasmid transfections (Fig. 6a–d) were performed on three different days (independent biological replicates) with two to three wells per assay (technical replicates). PCR experiments (Fig. 5e and Extended Data Fig. 1a) were performed at least twice with on-target amplicons confirmed by capillary sequencing.

### Reporting summary

Further information on research design is available in the Nature Portfolio Reporting Summary linked to this article.

## Online content

Any methods, additional references, Nature Portfolio reporting summaries, source data, extended data, supplementary information, acknowledgements, peer review information; details of author contributions and competing interests; and statements of data and code availability are available at 10.1038/s41593-024-01650-2.

### Supplementary information

Supplementary Information Supplementary Methods, References, Figs. 1–4 and Table 4.

Reporting Summary

Supplementary Table Supplementary Tables 1–3.

Supplementary Data Source data for Supplementary Figs. 1–4.

### Source data

Source Data Fig. 1 Statistical source data.

Source Data Fig. 1 Uncropped gel image.

Source Data Fig. 2 Statistical source data.

Source Data Fig. 3 Statistical source data.

Source Data Fig. 4 Statistical source data.

Source Data Fig. 5 Statistical source data.

Source Data Fig. 5 Uncropped gel image.

Source Data Fig. 6 Statistical source data.

Source Data Extended Data Fig. 1 Uncropped gel image.

Source Data Extended Data Fig. 5 Statistical source data.

Source Data Extended Data Fig. 6 Statistical source data.

Source Data Extended Data Fig. 8 Statistical source data.

Source Data Extended Data Fig. 9 Statistical source data.

## Data Availability

ONT sequencing data (.fastq and.fast5) generated from sorted hippocampal cell populations are available from the European Nucleotide Archive (ENA) under accession number PRJEB47835, as are PacBio L1 5′RACE data generated from bulk mouse hippocampus and sorted PV interneurons. The SOX6 consensus motif was downloaded from JASPAR (https://jaspar.elixir.no/), identifier MA0515.1. The L1 T_FI_ consensus sequence was obtained from RepBase (https://www.girinst.org/repbase/) version 18.03. Ontologies of genes containing intronic L1s were assessed with PANTHER (https://www.pantherdb.org/). Published PacBio long-read transcriptome sequencing data of adult mouse hippocampus tissue was obtained from ENCODE (https://www.encodeproject.org/), library identifier ENCLB505CBY. Retrotransposon genomic coordinates were downloaded from the UCSC Genome Browser RepeatMasker track (https://genome.ucsc.edu/cgi-bin/hgTables). ENCODE SOX6 and YY1 chromatin immunoprecipitation followed by sequencing (ChIP-seq) binding profiles over the L1Hs 5′UTR were generated by MapRRCon (http://maprrcon.org/). Human hippocampus single-cell ATAC-seq data were obtained from the Sequence Read Archive (SRA) under identifiers SRR11442501 and SRR11442502. Mouse cortex ATAC-seq and RNA sequencing (RNA-seq) data generated from excitatory pyramidal neuron, PV interneuron and VIP interneuron nuclei were obtained from the SRA (ATAC-seq: identifiers SRR1647880-SRR1647885, RNA-seq: identifiers SRR1647854-SRR1647859). Bulk hippocampus RNA-seq obtained from wild-type and conditional *Ctcf*-knockout animals were downloaded from the SRA (identifier SRP078142), as were RNA-seq data from neurons differentiated in vitro from human induced pluripotent stem cells, with and without LHX6 overexpression (SRA identifier SRP147748). L1.3 retrotransposition reporter and LRE3 promoter reporter constructs carrying mutant SOX binding sites, as well as cL1_spa_ reporter constructs, are available from the last author on request. Source data are provided with this paper.

## Code availability

Nanopore methylation analyses were performed with MethylArtist (https://github.com/adamewing/methylartist). Bisulfite sequencing results were visualized with QUMA (http://quma.cdb.riken.jp/). RNA-seq and ATAC-seq datasets were analyzed by pipelines joining together in serial published bioinformatic tools (see Methods). A custom Python script (https://gist.github.com/adamewing/331e1780975969afcebc2996ddb8c7a2) was written to analyze single-cell ATAC-seq profiles of the L1Hs promoter.
