# Supplementary material for: LINE-1 retrotransposons contribute to mouse PV interneuron development
Source: Nat Neurosci. 2024 May 21;27(7):1274–84. doi: 10.1038/s41593-024-01650-2 (PMC11239520; doi:10.1038/s41593-024-01650-2)
Supplement: Supplementary file 1 — Supplementary Methods, References, Figs. 1–4 and Table 4. [file 41593_2024_1650_MOESM1_ESM.pdf]

nature neuroscience

Supplementary information

https://doi.org/10.1038/s41593-024-01650-2

LINE-1 retrotransposons contribute to mouse PV interneuron development

In the format provided by the authors and unedited

Supplementary Methods

Cell sorting and nucleic acid isolation

Neonate litters were obtained from time-mated C57BL/6 mice bred in-house at the QBI animal facility. The day of birth was defined as postnatal day 0 (P0). From each P0 litter of ~6 pups we dissected and pooled hippocampus tissue. Tissues were dissociated in a papain solution, containing approximately 20U papain (Worthington) and 0.025mg DNase I (Worthington). Prior to use, papain was dissolved in HBSS (Gibco) with 1.1mM EDTA (Invitrogen), 0.067mM mercaptoethanol (Sigma) and 5mM cysteine-HCL (Sigma), and diluted in Hibernate E medium (Gibco). Tissue was incubated for 10min at 37°C with 0.5mL papain solution per embryo. Following digestion, the cell suspension was passed through a 70μm mesh cell strainer, washed into Hibernate E supplemented with B27 (Gibco) and then centrifuged at 300g for 5min. From this point in the protocol onwards, reagents were pre-chilled and the remaining procedures performed on ice. The cell pellet was resuspended in a blocking buffer (HBSS with 5% BSA). A rabbit anti-PV conjugated Alexa Fluor 647 antibody (Bioss bs-1299R-A647, dilution 1:2000) was directly added to the blocking buffer cell suspension and incubated for 1h at 4°C, then passed through a 40μm mesh cell strainer and subjected to flow cytometry. The cell suspension was run through a 100μm nozzle at low pressure (28psi) on a BD FACSAria II flow cytometer (Becton Dickinson). This first sort isolated PV^+ and PV^- cells. To further isolate PV^- neurons, PV^- cells from the first sort were collected in tubes containing 40U RNAseOUT ribonuclease inhibitor (Invitrogen), then fixed in ice cold 50% ethanol for 5min and centrifuged at 300g for 7min. Following centrifugation, cells were immunostained in blocking buffer containing mouse anti-beta III Tubulin (Tub) conjugated Alexa Fluor 488 antibody (Abcam ab195879, dilution 1:1000) and DAPI (Sigma D9542, 1μg/mL) for 15min at 4°C. Tub^+ immunostained cells were subjected to a second sort on the same FACS machine and specification as above. Four populations of cells were collected: PV^+ and PV^- (Supplementary Fig. 1a**,** sort 1) and PV^-/Tub^+ and PV^-/Tub^- (Supplementary Fig. 1a**,** sort 2). DNA and RNA were then extracted from each cell population. For RNA extractions, cells were sorted directly into the lysis buffer provided in the NucleoSpin RNA XS kit (Macherey Nagel), with RNA extraction performed following the manufacturer's specifications, except DNAse treatment was performed on a column twice for 20min, instead of once for 15min. For DNA extraction, purified cells were collected into a DNA lysis buffer containing TE buffer (10mM Tris-HCl pH 8 and 0.1mM EDTA), 2% SDS and 100μg/mL proteinase K, and DNA was extracted

following a standard phenol-chloroform protocol.

Caps2.L1 5'RACE and RT-PCR

For 5′RACE, hippocampus tissue from three adult C57BL/6 mice was pooled and RNA extracted (Trizol reagent, Invitrogen Cat# 15596026). RNA was used as input for a FirstChoice RLM-RACE Kit (Invitrogen, Cat# AM1700) to generate cDNA from capped, full-length mRNAs, following the manufacturer's specifications. Total RNA extracted from purified PV^+, PV^- and pooled neonate hippocampi was reverse transcribed using a High-Capacity cDNA Reverse Transcription Kit (Invitrogen, Cat# 4368814). PCR amplification was then performed using 1U MyTaq HS DNA Polymerase (BioLine) in 1× MyTaq buffer, 10pmol primer Caps2.L1_F (Supplementary Table 4), 10pmol primer Caps2.L1_R, 1µL cDNA in a 20µL final volume reaction. PCR cycling conditions were as follows: 95°C for 1min, (95°C for 15sec; 55°C for 15sec; 72°C for 10sec)×38, 72°C for 5min. Reaction products were run on a 1.5% agarose gel in 1×TAE, stained with SYBR Safe DNA gel stain.

L1 T_F promoter bisulfite sequencing

Targeted bisulfite sequencing was performed as described previously[54] to assess L1 T_F 5′UTR monomer CpG methylation genome-wide. Briefly, this involved extraction of genomic DNA from PV^+, PV^- and PV^-/Tub^+ populations purified from hippocampus tissue pooled from neonate littermates (Supplementary Fig. 1). Approximately 4×10^4 events per population were obtained from each of 3 litters (experimental triplicates). DNA was extracted via a conventional phenol-chloroform method and ethanol precipitation aided by glycogen (Ambion). DNA concentration was assessed with a Qubit dsDNA HS assay kit. Next, 20ng of genomic DNA was bisulfite converted using the EZ-DNA Methylation Lightning kit (Zymo Research, Cat# D5030) following the manufacturer's specifications. Bisulfite PCR reactions used MyTaq HS DNA polymerase (Bioline), and contained 1× reaction buffer, 12.5pmol of each primer, 2µL bisulfite treated DNA input template and 1U of enzyme in a 25µL final volume. PCR cycling conditions were as follows: 95°C for 2min, followed by 40 cycles of 95°C, 30sec; 54°C, 30sec; 72°C, 30sec and 1 cycle of 72°C, 5min. Primer sequences (BS_L1_TF_F and BS_L1_TF_R) were as provided in Supplementary Table 4. PCR products were visualized by electrophoresis on a 2% agarose gel, followed by the excision of fragments of expected size and DNA extracted using a MinElute gel

extraction kit (Qiagen, Cat# 28604) following the manufacturer's specifications. DNA concentration was assessed with a Qubit dsDNA HS assay kit and 30ng converted DNA was used as input for library preparation. Libraries were prepared using a NEBNext Ultra II DNA library prep kit (NEB, Cat# E7645S) and NEBNext Multiplex Oligos for Illumina (NEB, Cat# E6609S). Libraries were eluted in 15µL H_20 and concentrations measured with an Agilent 2100 Bioanalyzer using an Agilent HS DNA kit (Agilent Technologies, Cat# 5067-4627). Barcoded libraries of PV^+, PV^- and PV^-/Tub^+ populations from each of the 3 litters were mixed in equimolar quantities, diluted to 8nM, and combined with 50% PhiX spike-in control (Illumina, Cat# FC-110-3001). Single-end 300mer sequencing was then performed on a MiSeq platform (Illumina) using a MiSeq Reagent v3 kit (Illumina, Cat# MS-102-3003). Data were then analyzed as described elsewhere[17]. To summarize, reads with the L1 T_F bisulfite PCR primers at their termini were retained and aligned to the mock converted T_F monomer target amplicon sequence with blastn. Reads where non-CpG cytosine bisulfite conversion was <95%, or ≥5% of CpG dinucleotides were mutated, or ≥5% of adenine and guanine nucleotides were mutated, were removed. 100 reads per triplicate cell population, excluding identical bisulfite sequences, were randomly selected and analyzed using QUMA[73] version 1.1.16 with default parameters, with strict CpG recognition.

In utero *electroporation*

Embryonic *in utero* electroporation was employed to simultaneously deliver control (pmCherry) and experimental (L1) plasmids. Here, pmCherry *w*as a 4.7kb plasmid that expressed mCherry fluorescent protein under the control of a CMV promoter (Addgene, Cat# 632524). L1 plasmids consisted of pUBC-L1SM-UBC-EGFP and pMut2-UBC-L1SM-UBC-EGFP. pUBC-L1SM-UBC-EGFP was a derivative of cep99-GFP-L1SM, which contained a full-length codon-optimized synthetic mouse L1 T_F element (L1SM, kindly shared by Jef Boeke, NYU Langone)[47], where mouse ubiquitin C (UBC) promoters were substituted for the CMV promoters used to drive L1SM and EGFP expression in cep99-GFP-L1SM. pMut2-UBC-L1SM-UBC-EGFP was identical to pUBC-L1SM-UBC-EGFP, apart from two non-synonymous mutations in the L1SM ORF2 sequence known to disable ORF2p reverse transcriptase and endonuclease activities. *In utero* electroporation was performed as described previously[74], with the day of mating defined as embryonic day 0 (E0). Briefly, time-mated pregnant CD1 mice were anesthetized at E14.5 via an intraperitoneal injection of ketamine/xylazine (120mg/kg ketamine and 10mg/kg xylazine).

Embryos were exposed via a laparotomy and 0.5-1.0μL of plasmid DNA combined with 0.0025% Fast Green dye, to aid visualization, was injected into the lateral ventricle of each embryo using a glass-pulled pipette connected to a Picospritzer II (Parker Hannifin). Injections involved either combinations of pUBC-L1SM-UBC-EGFP and pmCherry (1μg/μL each) or pMut2-UBC-L1SM-UBC-EGFP and pmCherry (1μg/μL each). Half of the pups from each litter were co-injected with pUBC-L1SM-UBC-EGFP and pmCherry into the left hemisphere and the other half with pMut2-UBC-L1SM-UBC-EGFP and pmCherry into the right hemisphere. Plasmids were directed into the forebrain by placement of 3mm diameter microelectrodes across the head, which delivered 5 (100ms, 1Hz) approximately 36V square wave pulses via an ECM 830 electroporator (BTX). Once embryos were electroporated, uterine horns were replaced inside the abdominal cavity and the incision sutured closed. Dams received 1mL of Ringer's solution subcutaneously and an edible buprenorphine gel pack for pain relief. Dams were monitored daily until giving birth to live pups, which were collected for analysis at P10. All procedures were followed as approved by the University of Queensland Animal Ethics Committee (MRI-UQ/QBI/415/17).

Quantitative PCR on sorted cells and bulk hippocampus

Total RNA extracted from purified PV^+, PV^-, PV^-/Tub^+ and PV^-/Tub^- (Supplementary Fig. 1a, sorts 1 and 2) populations was used as input for SYBR Green and TaqMan qPCR assays. qPCR reactions were carried out using 300pg RNA/μL from purified PV^+ and PV^- cells and 100pg RNA/μL from purified PV^-/Tub^+ and PV^-/Tub^- cells. An RNA integrity number (RIN) above 6, as measured on an Agilent Bioanalyzer (Agilent Technologies, RNA 6000 Pico Kit, Cat# 5067-1513), was set as the minimum cutoff for RNA quality. All qPCRs were carried out on a LightCycler 480 Real-Time PCR system (Roche Life Science). Oligonucleotide PCR primers, as listed in Supplementary Table 4, were purchased from Integrated DNA Technologies. *SYBR Green assay*: PCR reactions were prepared using the Power SYBR Green RNA-to-CT 1 step kit (Applied Biosystems, Cat# 4391112). Reactions contained a 2× Power SYBR Green RT-PCR Mix, 10pmol of each primer, 1μL RNA input template and 1× reverse transcriptase enzyme mix in a 10μL final volume. Cycling conditions were as follows: 48°C for 30min, 95°C for 10min, followed by 40 cycles of 95°C, 15sec; 60°C, 1min. To assess potential DNA contamination, an L1 T_F qPCR using primers L1Md_5UTR_F and L1Md_5UTR_R was performed with and without reverse transcriptase. A three or more cycle difference between experiments run with and without reverse transcriptase,

and detection after cycle 30 in the latter, was considered as non-DNA contaminated RNA. *TaqMan assay*: Applied Biosystems custom L1, URR1 and 5S rRNA TaqMan MGB probes, as listed in Supplementary Table 4, were purchased from Thermo Fisher (Cat# 4316032), as was a proprietary mouse *Gapdh* combination (Cat# 4352339E). TaqMan qPCR reactions contained: 4× TaqPath 1-Step RT-qPCR multiplex reaction master mix (ThermoFisher, Cat# A28521), 4pmol of each primer, 1pmol probe (with the exception of the ORF2/URR1 TaqMan reaction, for which we used 1pmol ORF2 primers) and 1µL RNA input template in a 10uL final volume. Cycling conditions were as follows: 37°C for 2min; 50°C for 15min; 95°C for 2min, followed by 40 cycles of 95°C, 3sec; 60°C, 30sec. TaqMan assays for L1 were multiplexed with assays for either 5S rRNA, *Gapdh* or URR1 controls. L1 probes were conjugated to VIC or 6FAM fluorophores. Controls were conjugated to HEX, VIC or 6FAM fluorophores. Primer/probe sequences and the associated detection channels are listed in Supplementary Table 4. For each assay, the relative mRNA expression in a particular sample was calculated by the delta delta-CT method, using the negative population in the respective sort as control, i.e. PV^+ was compared to PV^- (Supplementary Fig. 1a, sort 1) and PV^-/Tub^+ compared to PV^-/Tub^- (Supplementary Fig. 1a, sort 2). As the PV^-/Tub^+ and PV^-/Tub^- populations were isolated as a result of two sortings in serial, for some assays sufficient RNA was only available to perform qPCR on PV^+ and PV^- populations. For qPCR on bulk hippocampus, tissue was isolated from 12-week old animals housed in standard (STD, *N*=12) and enriched (ENR, *N*=14) environments. RNA extraction was performed by Trizol following the manufacturer’s specifications (Trizol reagent, Invitrogen Cat# 15596026). Quantitative TaqMan PCR assays were performed as described above, using 40ng of RNA as input.

RNA-seq analysis

The mappability of individual TE copies generally varies as a function of sequencing read length, as well as TE subfamily age and copy number[75,76]. We therefore adopted a prior approach to quantify young mouse (L1 T_F) and human (L1Hs) subfamily-level transcript abundance with RNA-seq[20,75,77]. Analyzed datasets included Sams *et al.*[51], bulk hippocampus single-end (1×61mer) RNA-seq obtained from wild-type and conditional *Ctcf* knockout animals (SRA: SRP078142, *N*=3 pools of 2 animals per group), and Yuan *et al.*[50] bulk single-end (1×49mer) RNA-seq of neurons differentiated *in vitro* from human induced pluripotent stem cells, with and without LHX6 overexpression (SRA: SRP147748, *N*=3 per group). For each RNA-seq library, we

aligned reads to the reference genome (mouse: mm10, human: hg38) genome assembly with STAR[78] version 2.6 (parameters --twopassMode Basic --outSAMprimaryFlag AllBestScore --winAnchorMultimapNmax 1000 --outFilterMultimapNmax 1000) and marked duplicate reads with Picard MarkDuplicates (http://broadinstitute.github.io/picard). We expected the high copy number and limited divergence of young L1 subfamilies to cause most of the corresponding RNA-seq reads to "multi-map" to multiple genomic loci[75,76]. As conceived previously, we assigned multi-map reads a weighting at each of their aligned positions based on the abundance of uniquely mapping reads aligned within 100bp in the same library[20,75,77]. Each position was then assigned a weighting proportionate to the fraction of uniquely mapped reads found there, out of the total number of uniquely mapped reads within 100bp of any mapping position for the multi-mapping read. If no uniquely mapped reads were found near any of the aligned positions for a multi-mapped read, all positions were given an equal weighting. We then intersected the unique and weighted multi-map alignments with RepeatMasker coordinates and produced a total read count for L1 T_F (RepeatMasker: "L1Md_T") and L1Hs genome-wide, normalized by dividing by the total mapped read count for that RNA-seq library (tags-per-million).

Bulk ATAC-seq analysis

Mouse cortex ATAC-seq data were previously generated by Mo *et al.*[24] for excitatory pyramidal neurons (marked by Cam2ka), PV interneurons and VIP interneurons, via the isolation of nuclei tagged in specific cell types (INTACT) method. Paired-end fastq files were obtained from the Sequence Read Archive (SRA identifiers SRR1647880-SRR1647885). Trim Galore (parameters --max_n 2 --length 50 --trim-n) was used to apply CutAdapt[79] to read pairs to trim adapters and low quality bases. Processed reads were aligned to the reference genome (mm10) using bwa mem[80] with parameters (-a) to output all multimapping alignments. Alignments were filtered to keep only those with an alignment score equal to the maximum achieved for that read. The resulting bam files were sorted using samtools[67]. Peaks for each combined pair of duplicate experiments were called using MACS2[81] with default parameters, intersected with young L1 genomic coordinates, and then used to calculate the fraction of reads in each replicate aligned to at least one L1-associated peak.

scATAC-seq analyses

Human hippocampus scATAC-seq data reported by Corces *et al*.[49] were obtained from the SRA (identifiers SRR11442501 and SRR11442502). Read pairs were retained if the corresponding barcode was present in the 10x Genomics scATAC-seq Unique Molecular Identifier (UMI) whitelist (737K version 1) and then processed and aligned to the hg38 reference genome assembly, as per the bulk ATAC-seq analysis above. Cells (UMIs) with fewer than 10,000 uniquely aligned read pairs were discarded. For the human analysis, a cohort of 277 full-length (>5.9kbp) L1Hs elements defined previously[17] were employed. Cells were grouped into populations based on having at least one read aligned within the genomic coordinates of the proximal promoter of a given gene, with these coordinates as follows: PV, chr22:36816079-36818079; VIP, chr6:152749797-152751797; GFAP, chr17:44915750-44917750; EXC (CAMK2A), chr5:150289093-150291093. For each cell population, read depth was calculated across each full-length L1Hs copy, and these profiles were then summed to represent the L1Hs subfamily.

Environmental enrichment and exercise experimental design

At six weeks of age, CBA×C57BL/6 mice were randomly assigned to either a standard (STD), enriched environment (ENR) or exercise (EXE) group, as described previously[82]. All mice were exposed to their assigned housing condition for 6 weeks. Briefly, STD housing consisted of an open-top standard mouse cage (34 × 16 × 16cm; 4 mice/box) with basic bedding and nesting materials. ENR and EXE mice were housed in larger cages (40 × 28 × 18cm; 4 mice/box) containing the same basic bedding and nesting materials as the STD plus specific features. ENR cages contained climbing and tunneling objects together with inanimate objects of various textures, sizes, and shapes, which altogether confer the enhancement of sensory, cognitive and motor stimulation[83]. These cages were changed weekly to ensure novelty for ENR mice. In addition, from weeks 10-12, ENR mice were exposed three times a week for one hour to an extra ‘super-enriched’ condition in a larger playground arena (43 × 80 × 51cm) as previously described[84]. Each EXE cage contained two running wheels (12cm in diameter) to ensure mice had access to voluntary wheel running. Running wheels were excluded from the ENR housing to ensure the effects of physical activity were exclusive to the EXE mice. All mice had *ad libitum* access to food and water and were housed in a controlled room at 22°C and 45% humidity on a 12:12 hour light/dark cycle. All procedures were approved by The Florey Institute of Neuroscience and Mental Health Animal

Ethics Committee (19-012-FINMH) and were performed in accordance with the relevant guidelines and regulations of the Australian National Health and Medical Research Council Code of Practice for the Use of Animals for Scientific Purposes.

Supplementary References

73. Kumaki, Y., Oda, M. & Okano, M. QUMA: quantification tool for methylation analysis. *Nucleic Acids Res.* **36**, W170–5 (2008).
74. Paolino, A. *et al.* Differential timing of a conserved transcriptional network underlies divergent cortical projection routes across mammalian brain evolution. *Proc. Natl. Acad. Sci. U. S. A.* **117**, 10554–10564 (2020).
75. Faulkner, G. J. *et al.* A rescue strategy for multimapping short sequence tags refines surveys of transcriptional activity by CAGE. *Genomics* **91**, 281–288 (2008).
76. Lanciano, S. & Cristofari, G. Measuring and interpreting transposable element expression. *Nat. Rev. Genet.* **21**, 721–736 (2020).
77. Hashimoto, T. *et al.* Probabilistic resolution of multi-mapping reads in massively parallel sequencing data using MuMRescueLite. *Bioinformatics* **25**, 2613–2614 (2009).
78. Dobin, A. *et al.* STAR: ultrafast universal RNA-seq aligner. *Bioinformatics* **29**, 15–21 (2013).
79. Martin, M. Cutadapt removes adapter sequences from high-throughput sequencing reads. *EMBnet.journal* **17**, 10–12 (2011).
80. Li, H. Aligning sequence reads, clone sequences and assembly contigs with BWA-MEM. *arXiv [q-bio.GN]* arXiv:1303.3997 (2013).
81. Zhang, Y. *et al.* Model-based analysis of ChIP-Seq (MACS). *Genome Biol.* **9**, R137 (2008).
82. Love, C. J., Gubert, C., Renoir, T. & Hannan, A. J. Environmental enrichment and exercise housing protocols for mice. *STAR Protoc* **3**, 101689 (2022).
83. Gubert, C. & Hannan, A. J. Environmental enrichment as an experience-dependent modulator of social plasticity and cognition. *Brain Res.* **1717**, 1–14 (2019).
84. Mazarakis, N. K. *et al.* 'Super-Enrichment' Reveals Dose-Dependent Therapeutic Effects of Environmental Stimulation in a Transgenic Mouse Model of Huntington's Disease. *J. Huntingtons Dis.* **3**, 299–309 (2014).
85. Bedrosian, T. A., Quayle, C., Novaresi, N. & Gage, F. H. Early life experience drives

structural variation of neural genomes in mice. *Science* **359**, 1395–1399 (2018).

86. Bodea, L.-G. *et al.* Neurodegeneration by activation of the microglial complement-phagosome pathway. *J. Neurosci.* **34**, 8546–8556 (2014).

87. Filice, F., Vörckel, K. J., Sungur, A. Ö., Wöhr, M. & Schwaller, B. Reduction in parvalbumin expression not loss of the parvalbumin-expressing GABA interneuron subpopulation in genetic parvalbumin and shank mouse models of autism. *Mol. Brain* **9**, (2016).

88. O'Driscoll, C., Kaufmann, W. E. & Bressler, J. Relationship between Mecp2 and NFκb signaling during neural differentiation of P19 cells. *Brain Res.* **1490**, 35–42 (2013).

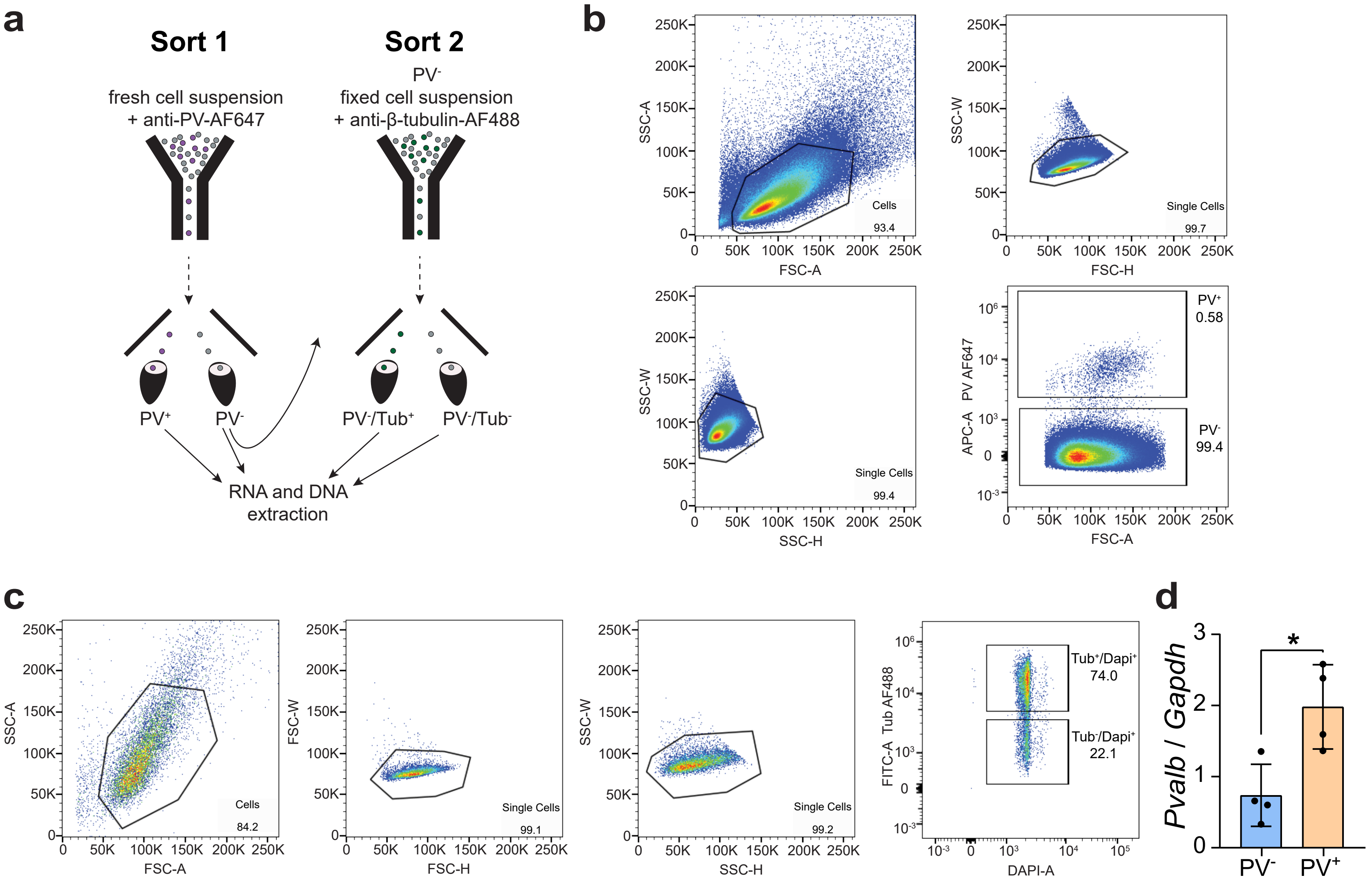

Supplementary Fig. 1: PV interneuron isolation. a, Schematic of PV$^+$ and PV$^-$/Tub$^+$ neuron isolation from pooled neonate (P0) litter hippocampal tissue. An anti-PV conjugated antibody (AF647) was used to label and isolate PV$^+$ cells. Freshly sorted PV$^-$ cells were subsequently labeled with an anti-Tub conjugated antibody (AF488) and sorted again to isolate PV$^-$/Tub$^+$ neurons. **b,** Gating strategy and purity of fluorescence activated cell sorting (FACS) for PV$^+$ cells. **c,** As for (b), except showing PV$^-$/Tub$^+$ neurons. **d,** Quality control qPCR of relative PV mRNA expression in sorted cells. *Pvalb* (parvalbumin) mRNA enrichment in the PV$^+$ population was observed, as expected. Data are relative to *Gapdh* and presented as mean ± SD. *P=0.015, two-tailed t test, N(litters)=4.

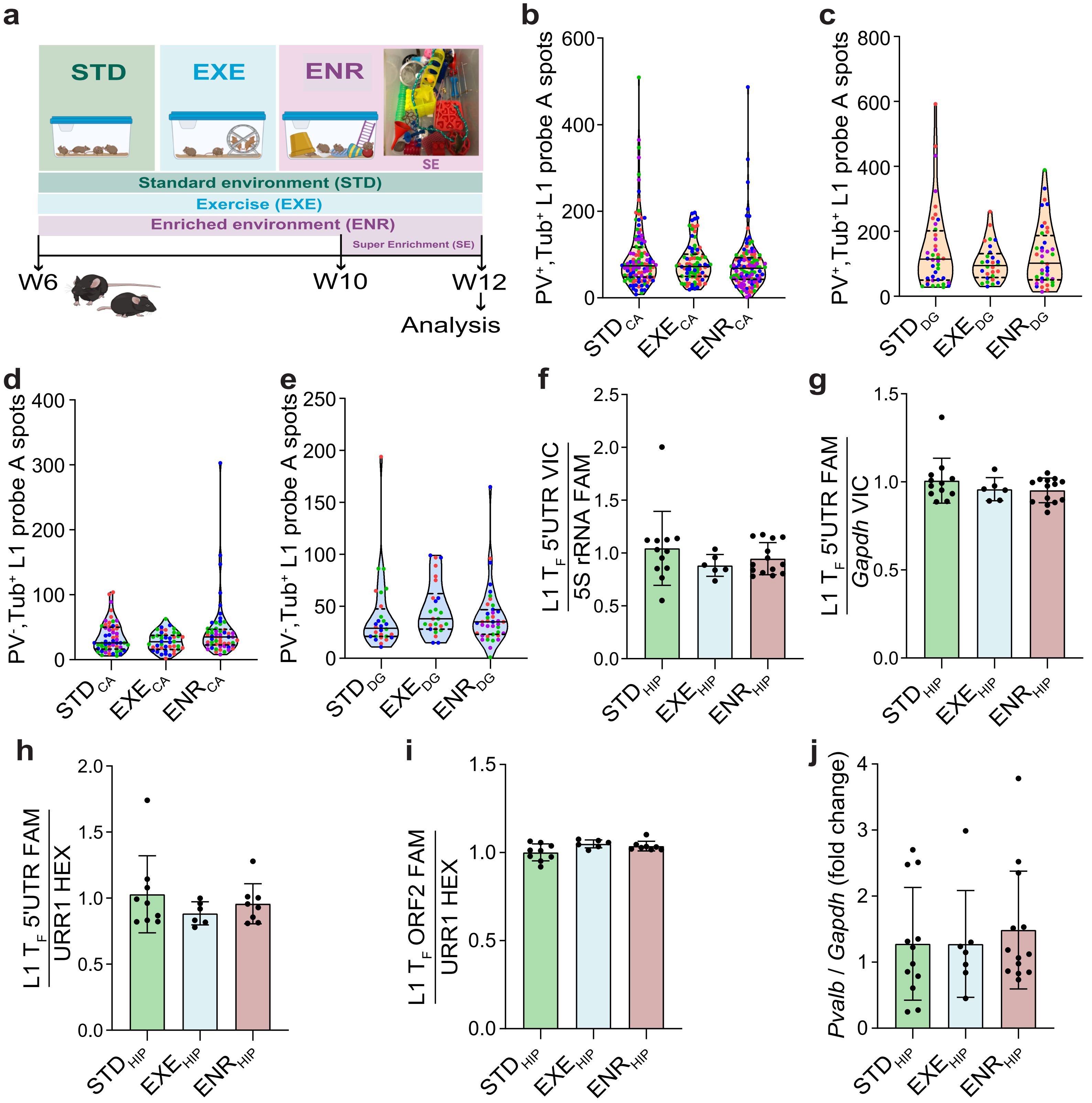

Supplementary Fig. 2: Environmental enrichment or exercise do not impact L1 mRNA abundance in PV interneurons. a, Standard (STD), exercise (EXE) and enriched (ENR) environment housing schematics. Mice (aged 6 weeks) were placed in either STD, EXE or ENR housing for 6 weeks. ENR and EXE housing consisted of a larger cage with nesting materials. EXE housing contained two running wheels to guarantee all mice had access to voluntary wheel running (excluded in the ENR group). ENR mice were also exposed to spatial stimuli; ladders, tunneling objects and toys of various textures, sizes, and shapes for sensory, cognitive and motor stimulation. Between week 10 and 12, ENR mice were exposed three times a week for one hour to ‘super-enriched’ condition in a larger playground arena with novel toys. **b,** L1 T_F RNA FISH spots (probe A) in PV^+/Tub^+ neurons from STD, EXE and ENR animal CA tissue. *N*(mice)=3-4. Cells from each mouse are color coded. Solid line: median. Dashed lines: quartiles. **c,** As for (b) but in DG. **d,** As per (b), except showing L1 T_F RNA FISH spots in PV^-/Tub^+ neurons. **e,** As for (d), except in DG. **f,** TaqMan qPCR measuring abundance of the L1 T_F mRNA monomeric 5′UTR (VIC channel) relative to 5S rRNA (FAM channel) in bulk hippocampus samples from STD, EXE and ENR mice. STD *N*=12, ENR *N*=14. **g,** As for (f), except targeting the L1 T_F non-monomeric 5′UTR (FAM channel) relative to *Gapdh* (VIC channel). **h,** As for (g), except measuring L1 T_F non-monomeric 5′UTR (FAM channel) relative to URR1 (HEX channel). **i,** As for (h) except targeting L1 T_F ORF2 (FAM channel) relative to URR1 (HEX channel). STD *N*=9, ENR *N*=8. **j,** *Pvalb* (parvalbumin) mRNA expression in STD, EXE and ENR conditions, relative to *Gapdh*. Note: values in (f-j) are represented as mean ± SD. Significance testing was via one-way ANOVA with Tukey’s post-hoc test comparing means of animals. No significant ($P<0.05$) differences were detected between groups.

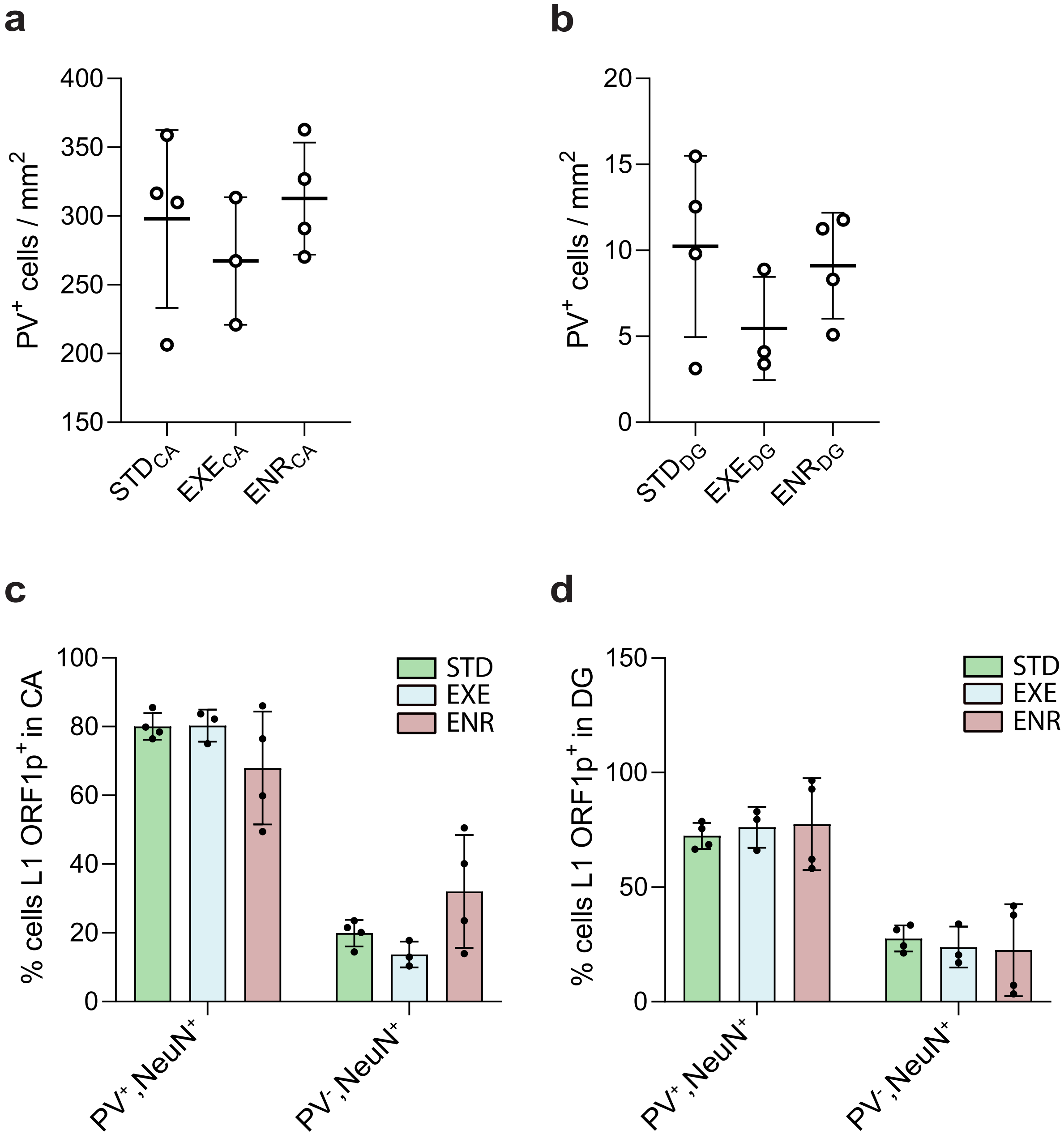

Supplementary Fig. 3: Environmental enrichment or exercise do not impact PV interneuron count or L1 ORF1p expression. a, Stereological estimation of PV$^+$ neuron number in CA of STD, EXE and ENR mice. N=3-4 mice per condition. **b,** As per (a), except for DG. **c,** Percentage of L1 ORF1p$^+$/PV$^+$ versus ORF1p$^+$/PV$^-$ neurons in STD, EXE and ENR mice. N=3-4 mice. **d,** As per (c), except for DG. Note: in (a) and (b) significance testing was via one-way ANOVA and in (c) and (d) via two-way ANOVA, each with Tukey's post-hoc test. No significant (P<0.05) differences were detected between groups. Data are represented as mean ± SD.

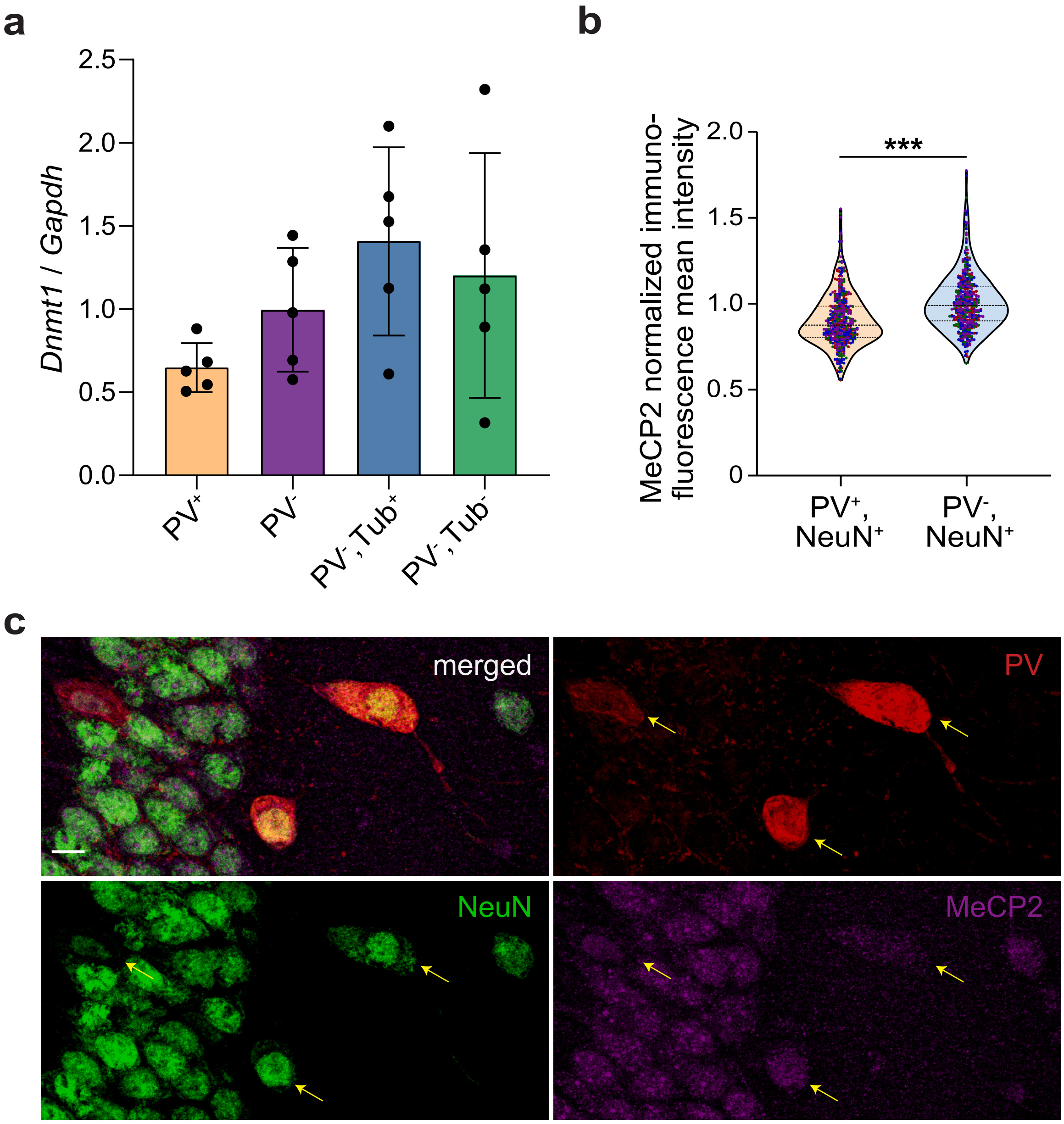

Supplementary Fig. 4: Relaxation of epigenetic repression in PV interneurons. a, *Dnmt1* mRNA abundance measured by qPCR in PV$^+$, PV$^-$, PV$^-$/Tub$^+$ and PV$^-$/Tub$^-$ cell populations, relative to *Gapdh*. Significance testing was via one-way ANOVA with Dunnett's multiple comparison test only to the PV$^+$ population, N=5 litters. Data are represented as mean ± SD. **b,** MeCP2 protein expression in PV$^-$/NeuN$^+$ (blue plot) and PV$^+$/NeuN$^+$ (orange plot) neurons. MeCP2 immunofluorescence mean intensities were obtained from coronal hippocampus sections stained for MeCP2, PV and NeuN, and normalized to the PV$^-$/NeuN$^+$ population mean. Two-tailed t test comparing mouse means, ***P=0.0002, n(cells)=103-104, N(mice)=4. Cells from each mouse are color coded. **c,** Representative immunostaining image of a coronal hippocampus section showing colocalization of MeCP2 (magenta) with PV (red) and the pan-neuronal marker NeuN (green). Yellow arrows indicate PV interneurons on single channel images. Scale bar: 10µm.

Supplementary Table 4. Primer and probe information.

**Name**	**Sequence**	**Target**	**Assay**	**Source**
BS_L1TFI/II_F	GTTGAGGTAGTATTTTGTGTGGGT	L1 $T_{FI/II}$ 5′UTR	Bisulfite PCR	[54]
BS_L1TFI/II_R	TTCCAAAAACTATCAAATTCTCTAAC	L1 $T_{FI/II}$ 5′UTR	Bisulfite PCR	[54]
URR1_F	GTAGCAAAATTATAGTTATGAAGTA GC	URR1	TaqMan qPCR	This study.
URR1_R	CTCAACCTTCCTAATGCT	URR1	TaqMan qPCR	This study.
URR1 probe	HEX-TCACCACAACATGAGGAACTGTATTA-MGBNFQ	URR1	TaqMan qPCR	This study.
L1TFnonmon_F	AGGTCCAAATACAAGATATCTGC	L1 T_F 5′UTR	TaqMan qPCR	This study.
L1TFnonmon_R	ATCAGCAGACCTGGGAGACA	L1 T_F 5′UTR	TaqMan qPCR	This study.
L1TFnonmon probe	FAM-TGCCAGCAGAGAGTGCTCTGAGC-MGBNFQ	L1 T_F 5′UTR	TaqMan qPCR	This study.
TaqORF2 Fw	CATCAATGTAATCCATTATATAAAC	L1 T_F ORF2	TaqMan qPCR	This study.
TaqORF2 Rv	TTTATCATGAATGGGTGTTG	L1 T_F ORF2	TaqMan qPCR	This study.
TaqORF2 probe	FAM-CACATGATCATCTCGTTAGATGCAGA-MGBNFQ	L1 T_F ORF2	TaqMan qPCR	This study.
Mouse *Gapdh* assay	Proprietary VIC®/MGB probe and primers	*Gapdh*	TaqMan qPCR	ThermoFisher
L1Md_5UTR_F	CAGCCGGCCACCTTCC	L1 $T_{FI/II}$ 5′UTR	TaqMan qPCR	[85]
L1Md_5UTR_R	GGTCCCGGACCAAGATGG	L1 $T_{FI/II}$ 5′UTR	TaqMan qPCR	[85]
L1Md_5UTR probe	VIC-CGGAGGACAGGTGC-MGBNFQ	L1 $T_{FI/II}$ 5′UTR	TaqMan qPCR	[85]
5S_rRNA_F	ACGGCCATACCACCCTGAA	5S rRNA	TaqMan qPCR	[85]
5S_rRNA_R	GGTCTCCCATCCAAGTACTAACCA	5S rRNA	TaqMan qPCR	[85]
5S_rRNA_probe	FAM-CCGAGATCAGACGAGAT-MGBNFQ	5S rRNA	TaqMan qPCR	[85]
Gapdh_F	AACTTTGGCATTGTGGAAGG	*Gapdh*	SYBR qPCR	[86]
Gapdh_R	GGATGCAGGGATGATGTTCT	*Gapdh*	SYBR qPCR	[86]
PV_F	TGTCGATGACAGACGTGCTC	*Pvalb*	SYBR qPCR	[87]
PV_R	TTCTTCAACCCCAATCTTGC	*Pvalb*	SYBR qPCR	[87]
MeCP2_F	AGGAGAGACTGGAGGAAAAGT	*Mecp2*	SYBR qPCR	[88]
MeCP2_R	CTTAAACTTCAGTGGCTTGTCT	*Mecp2*	SYBR qPCR	[88]
L1eGFP_6154	AATATCACGGGTAGCCAACG	EGFP	Genotyping	This study.
L1eGFP_7850	TAGCGCTACCGGACTCAGAT	EGFP	Genotyping	This study.
Caps2.L1_F	GTCACTCTTCCTGCCTGCTC	Caps2.L1	RACE/RT-PCR	This study.
Caps2.L1_R	AGTCAACAAGGCTTCCCAGA	Caps2.L1	RACE/RT-PCR	This study.
Dnmt1_F	CCTAGTTCCGTGGCTACGAGGAGAA	*Dnmt1*	SYBR qPCR	[55]
Dnmt1_R	TCTCTCTCCTCTGCAGCCGACTCA	*Dnmt1*	SYBR qPCR	[55]
Dnmt3a_F	GCCGAATTGTGTCTTGGTGGATGACA	*Dnmt3a*	SYBR qPCR	[55]
Dnmt3a_R	CCTGGTGGAATGCACTGCAGAAGGA	*Dnmt3a*	SYBR qPCR	[55]
5RACE_L1	CATCTCTTGTATTCTGTTGCTGATGCTCAA	L1 $T_{FI/II}$ ORF1	5′RACE	[25]
mCherry_F	GCGCAGCTTCACCTTGTAG	mCherry	Junction PCR	This study.
mCherry_R	GCGTGATGAACTTCGAGGAC	mCherry	Junction PCR	This study.
mSOX6_F	TTGGGGAGTACAAGCAACTGATGC	*Sox6*	SYBR qPCR	This study.
mSOX6_R	ATCTGAGGTGATGGTGTGGTCGTT	*Sox6*	SYBR qPCR	This study.
