# Supplementary material for: LINE-1 retrotransposons contribute to mouse PV interneuron development
Source: Nat Neurosci. 2024 May 21;27(7):1274–84. doi: 10.1038/s41593-024-01650-2 (PMC11239520; doi:10.1038/s41593-024-01650-2)
Supplement: Supplementary file 2 — Reporting Summary [file 41593_2024_1650_MOESM2_ESM.pdf]

nature portfolio

Corresponding author(s): Gabriela O. Bodea and Geoffrey J. Faulkner

Last updated by author(s): Mar 31, 2024

Reporting Summary

Nature Portfolio wishes to improve the reproducibility of the work that we publish. This form provides structure for consistency and transparency in reporting. For further information on Nature Portfolio policies, see our Editorial Policies and the Editorial Policy Checklist.

Statistics

For all statistical analyses, confirm that the following items are present in the figure legend, table legend, main text, or Methods section.

n/a	Confirmed	
☐	☒	The exact sample size (n) for each experimental group/condition, given as a discrete number and unit of measurement
☐	☒	A statement on whether measurements were taken from distinct samples or whether the same sample was measured repeatedly
☐	☒	The statistical test(s) used AND whether they are one- or two-sided<br>*Only common tests should be described solely by name; describe more complex techniques in the Methods section.*
☐	☒	A description of all covariates tested
☐	☒	A description of any assumptions or corrections, such as tests of normality and adjustment for multiple comparisons
☐	☒	A full description of the statistical parameters including central tendency (e.g. means) or other basic estimates (e.g. regression coefficient) AND variation (e.g. standard deviation) or associated estimates of uncertainty (e.g. confidence intervals)
☐	☒	For null hypothesis testing, the test statistic (e.g. F, t, r) with confidence intervals, effect sizes, degrees of freedom and P value noted<br>*Give P values as exact values whenever suitable.*
☒	☐	For Bayesian analysis, information on the choice of priors and Markov chain Monte Carlo settings
☒	☐	For hierarchical and complex designs, identification of the appropriate level for tests and full reporting of outcomes
☐	☒	Estimates of effect sizes (e.g. Cohen's d, Pearson's r), indicating how they were calculated

Our web collection on statistics for biologists contains articles on many of the points above.

Software and code

Policy information about availability of computer code

Data collection	Zen version 2009 (Zeiss)<br>CellSens Dimension software version 4.1.1 (Olympus)<br>SlideBook 6.0 (3I)<br>Stereo Investigator software version 2017.03.03 (MBF Bioscience)<br>FACSymphony™ A5 SE Cell Analyzer (BD Biosciences)<br>BD Accuri C6 Flow Cytometer software (BD Biosciences)<br>BD FACS Diva version 6.1.3 (BD Biosciences)
Data analysis	Fiji for Windows (ImageJ 1.52d)<br>Imaris version 9.5.1 (Bitplane, Oxford Instruments)<br>FlowJo (version 10.8.1, BD Biosciences)<br>Prism version 9.5.1 (GraphPad)<br>SciPy version 1.4.1 (https://scipy.org/)<br>SnapGene version 6.1.2 (GSL Biotech)<br>Methylartist version 1.1.1 (https://github.com/adamewing/methylartist)<br>QUMA version 1.1.16 (http://quma.cdb.riken.jp/)<br>STAR version 2.6 (https://github.com/alexdobin/STAR)<br>Picard MarkDuplicates version 2.27.5 (https://gatk.broadinstitute.org/hc/en-us)<br>bwa-mem version 0.7.12-r1039 (https://github.com/lh3/bwa)<br>CutAdapt version 3.4 (https://github.com/marcelm/cutadapt)<br>MACS2 version 2.2.7.1 (https://github.com/macs3-project/MACS)

minimap2 version 2.20 (https://github.com/lh3/minimap2)
SAMtools version 1.12 (https://github.com/samtools/samtools)
nanopolish version 0.13.2 (https://github.com/jts/nanopolish)
tabix version 1.12 (https://github.com/tabixio/tabix)
Guppy version 4.0.11 (Oxford Nanopore Technologies)
Custom Python script written to analyze scATAC-seq profiles of the L1Hs promoter (https://gist.github.com/adamewing/331e1780975969afcebc2996ddb8c7a2)

For manuscripts utilizing custom algorithms or software that are central to the research but not yet described in published literature, software must be made available to editors and reviewers. We strongly encourage code deposition in a community repository (e.g. GitHub). See the Nature Portfolio guidelines for submitting code & software for further information.

Data

Policy information about availability of data

All manuscripts must include a data availability statement. This statement should provide the following information, where applicable:

- Accession codes, unique identifiers, or web links for publicly available datasets
- A description of any restrictions on data availability
- For clinical datasets or third party data, please ensure that the statement adheres to our policy

ONT sequencing data (.fastq and .fast5) generated from sorted hippocampal cell populations are available from the European Nucleotide Archive (ENA) under accession number PRJEB47835. PacBio L1 5′RACE data generated from bulk mouse hippocampus and sorted PV interneurons are also available from PRJEB47835.
The SOX6 consensus motif was downloaded from JASPAR (https://jaspar.elixir.no/), identifier MA0515.1.
The L1 TFI consensus sequence was obtained from RepBase (https://www.girinst.org/repbase/) version 18.03.
Ontologies of genes containing intronic L1s were assessed with PANTHER (https://www.pantherdb.org/).
Published PacBio long-read transcriptome sequencing data of adult mouse hippocampus tissue was obtained from ENCODE (https://www.encodeproject.org/), library identifier ENCLB505CBY.
Retrotransposon genomic coordinates were downloaded from the UCSC Genome Browser RepeatMasker track (https://genome.ucsc.edu/cgi-bin/hgTables).
ENCODE SOX6 and YY1 ChIP-seq binding profiles over the L1Hs 5′UTR were generated by MapRRCon (http://maprrcon.org/).
Human hippocampus scATAC-seq data were obtained from the Sequence Read Archive (SRA) under identifiers SRR11442501 and SRR11442502.
Mouse cortex ATAC-seq and RNA-seq data generated from excitatory pyramidal neuron, PV interneuron and VIP interneuron nuclei were obtained from the SRA (ATAC-seq: identifiers SRR1647880-SRR1647885, RNA-seq: identifiers SRR1647854-SRR1647859).
Bulk hippocampus RNA-seq obtained from wild-type and conditional CTCF knockout animals was downloaded from the SRA (identifier SRP078142).
RNA-seq data from neurons differentiated in vitro from human induced pluripotent stem cells, with and without LHX6 overexpression, was downloaded from the SRA (identifier SRP147748).

Research involving human participants, their data, or biological material

Policy information about studies with human participants or human data. See also policy information about sex, gender (identity/presentation), and sexual orientation and race, ethnicity and racism.

Reporting on sex and gender	N/A
Reporting on race, ethnicity, or other socially relevant groupings	N/A
Population characteristics	N/A
Recruitment	N/A
Ethics oversight	N/A

Note that full information on the approval of the study protocol must also be provided in the manuscript.

Field-specific reporting

Please select the one below that is the best fit for your research. If you are not sure, read the appropriate sections before making your selection.

☒ Life sciences ☐ Behavioural & social sciences ☐ Ecological, evolutionary & environmental sciences

For a reference copy of the document with all sections, see nature.com/documents/nr-reporting-summary-flat.pdf

Life sciences study design

All studies must disclose on these points even when the disclosure is negative.

Sample size	Animal experiments were conducted with, at a minimum, 3 biological replicates. These replicates consisted of either individual animals or pooled material. These minimum sample sizes were selected based on common conventions in the field to enable robust statistical analyses considering practical limitations on time/resources/cost. Some meta-analyses of published data (e.g. RNA-seq or ATAC-seq) involved

	duplicates rather than triplicates. Information about the number of animals or cells used in each experiment can be found in the figure legends.
Data exclusions	No data were excluded.
Replication	Experiments were conducted with biological replicates and technical replicates. Statistical significance was determined based on biological replicates (as outlined in the Sample Size section above). The most unexpected finding was the hypomethylation of L1 promoters in PV neurons. We first identified this with bisulfite sequencing, and then replicated this result with nanopore sequencing, a different approach using different input material.
Randomization	We used stratified randomisation based on age to allocate mice to experimental groups. The cell culture experiments were not randomized.
Blinding	Blinding to group allocation was used for image analyses involving different treatment groups (Fig. 6a-d, Extended Data Fig. 9f, Supplementary Fig. 2, Supplementary Fig. 3). No blinding to sample identity during sample collection was used.

Reporting for specific materials, systems and methods

We require information from authors about some types of materials, experimental systems and methods used in many studies. Here, indicate whether each material, system or method listed is relevant to your study. If you are not sure if a list item applies to your research, read the appropriate section before selecting a response.

Materials & experimental systems

n/a	Involved in the study
☐	☒ Antibodies
☐	☒ Eukaryotic cell lines
☒	☐ Palaeontology and archaeology
☐	☒ Animals and other organisms
☒	☐ Clinical data
☒	☐ Dual use research of concern
☒	☐ Plants

Methods

n/a	Involved in the study
☒	☐ ChIP-seq
☐	☒ Flow cytometry
☒	☐ MRI-based neuroimaging

Antibodies

Antibodies used

Rabbit anti-GFP (Thermo Fisher A11122)
Chicken anti-GFP (Millipore AB16901)
Mouse anti-T7 (Millipore 69522)
Rabbit anti-T7 (Millipore AB3790)
Goat anti-tdTomato (Sicgen AB8181)
Mouse anti-NeuN, clone A60 (Millipore MAB377)
Guinea pig anti-NeuN (Millipore ABN90)
Rabbit anti-parvalbumin (PV) conjugated Alexa Fluor 647 (Bioss bs-1299R-A647)
Mouse anti-beta III Tubulin conjugated Alexa Fluor 488 (Abcam ab195879)
Rabbit anti-Gad65/67 (GAD1) (Sigma G5163)
Mouse anti-parvalbumin (PV) (Sigma P3088)
Rabbit anti-β tubulin III (Tub) (Sigma T2200)
Mouse anti-β-tubulin III (Tub) (Sigma, T4026)
Rabbit anti-MeCP2 (Abcam ab2828)
Rabbit anti-Cre (Cell Signaling, 15036)
Rabbit anti-parvalbumin (PV) (Swant, PV27)
Rabbit anti-ORF1p (Abcam, ab216324)
Donkey anti-guinea pig Dylight 405 (Jackson Immunoresearch 706475148)
Donkey anti-mouse Dylight 405 (Jackson Immunoresearch 715475150)
Donkey anti-chicken Alexa Fluor 488 (Jackson Immunoresearch 703546155)
Donkey anti-rat Alexa Fluor 488 (Jackson Immunoresearch 712546150)
Donkey anti-rabbit Alexa Fluor 488 (Thermo Fisher A21206)
Donkey anti-goat Alexa Fluor 594 (Jackson Immunoresearch 705586147)
Donkey anti-goat Cy3 (Jackson ImmunoResearch 715-165-150)
Donkey anti-rabbit Cy3 (Jackson Immunoresearch 711165152)
Donkey anti-mouse Cy3 (Jackson Immunoresearch 715165150)
Donkey anti-guinea pig Alexa Fluor 647 (Millipore AP193SA6)
Donkey anti-rabbit Alexa Fluor 647 (Jackson ImmunoResearch)

Validation

Rabbit anti-GFP (Thermo Fisher A11122), verified by manufacturer for relative expression to ensure binding to the antigen stated and previously used for the same application (e.g. PMID: 34772739, 32152287).
Chicken anti-GFP (Millipore AB16901), previously used for the same species and application (PMID:24804730, 19025635).
Mouse anti-T7 (Millipore 69522), previously used for the same application (PMID: 8033208, 7637813).
Rabbit anti-T7 (Millipore AB3790), previously used for the same application (PMID: 37741199).
Goat anti-tdTomato (Sicgen AB8181), previously used for the same application (PMID: 37775311).

Mouse anti-NeuN, clone A60 (Millipore MAB377), manufacturer validated for the application and published (PMID: 9545178).
Guinea pig anti-NeuN (Millipore ABN90), manufacturer validated for the application and published (PMID: 34761053).
Rabbit anti-parvalbumin (PV) conjugated Alexa Fluor 647 (Bioss bs-1299R-A647), manufacturer validated for other applications. Data for flow cytometry application provided in the manuscript (Supplementary Fig. 1).
Mouse anti-beta III Tubulin conjugated Alexa Fluor 488 (Abcam ab195879), knockout validated by the manufacturer.
Rabbit anti-Gad65/67 (GAD1) (Sigma G5163), manufacturer validated for the application and published (PMID: 25093893).
Mouse anti-parvalbumin (PV) (Sigma P3088), manufacturer validated for the application and published (PMID:25299405)
Rabbit anti-β tubulin III (Tub) (Sigma T2200), knockout validated by the manufacturer.
Mouse anti-β-tubulin III (Tub) (Sigma, T4026), previously used for the same application (PMID:11425343)
Rabbit anti-MeCP2 (Abcam ab2828), manufacturer validated for the application and species, cited in >75 publications.
Rabbit anti-Cre (Cell Signaling, 15036), previously used for the same species and application (PMID: 37239343)
Rabbit anti-parvalbumin (PV) (Swant, PV27), previously used for the same species and application (PMID: 28384468)
Rabbit anti-ORF1p (Abcam, ab216324), manufacturer validated for the application and species. Data provided in the manuscript (Extended Data Fig. 7).
All Jackson Immunoresearch secondary antibodies, manufacturer tested by ELISA and/or solid-phase adsorbed to ensure minimal cross-reaction with other species.
Donkey anti-rabbit Alexa Fluor 488 (Thermo Fisher A21206), tested by individual users and used in >6000 publications.
Donkey anti-guinea pig Alexa Fluor 647 (Millipore AP193SA6), previously used for the same application (e.g. PMID: 36512397)

Eukaryotic cell lines

Policy information about cell lines and Sex and Gender in Research

Cell line source(s)	PA-1 and N2a cells were obtained from the ATCC. HeLa-JVM (a subtype of HeLa) cells were obtained from the laboratory of John V. Moran.
Authentication	None of the cell lines were authenticated.
Mycoplasma contamination	Cells were tested for mycoplasma contamination and returned a negative result.
Commonly misidentified lines (See ICLAC register)	No commonly misidentified cell lines were used in the study.

Animals and other research organisms

Policy information about studies involving animals; ARRIVE guidelines recommended for reporting animal research, and Sex and Gender in Research

Laboratory animals	L1-EGFP animals were C57BL/6 and analysed at 12 weeks age. RNAscope and flow cytometry experiments were conducted on neonate C57BL/6 animals. Primary neuronal cultures were obtained from E18 C57BL/6 embryos. In utero electroporation experiments were conducted on CD1 animals, and pups analysed at P10. Environmental enrichment and exercise experiments were conducted on CBA×C57BL/6 animals from 6 weeks of age.
Wild animals	The study did not involve wild animals.
Reporting on sex	Male and female mice were used in approximately equal numbers for each experiment, but the N per sex was underpowered to examine sex differences. Sex was therefore not included as a factor in statistical analyses.
Field-collected samples	The study did not involve samples collected from the field.
Ethics oversight	All procedures were followed as approved by the University of Queensland Animal Ethics Committee (TRI/UQ-MRI/381/14/NHMRC/DFG and MRI-UQ/QBI/415/17) and the Florey Institute of Neuroscience and Mental Health Animal Ethics Committee (19-012-FINMH).

Note that full information on the approval of the study protocol must also be provided in the manuscript.

Plants

Seed stocks	N/A
Novel plant genotypes	N/A
Authentication	N/A

Flow Cytometry

Plots

Confirm that:

☒ The axis labels state the marker and fluorochrome used (e.g. CD4-FITC).

☒ The axis scales are clearly visible. Include numbers along axes only for bottom left plot of group (a 'group' is an analysis of identical markers).

☒ All plots are contour plots with outliers or pseudocolor plots.

☒ A numerical value for number of cells or percentage (with statistics) is provided.

Methodology

Sample preparation	C57BL/6 neonate hippocampi were dissociated, pelleted, resuspended in DPBS and immunostained for parvalbumin. Sorted parvalbumin negative cells were fixed and immunostained for beta III Tubulin.
Instrument	BD FACS Aria I (Becton Dickinson)
Software	Data were collected and analysed using BD FACS Diva 6.1.3 and FlowJo software.
Cell population abundance	In the starting population, parvalbumin positive population represented ~ 0.5%. Within the parvalbumin negative population ~ 70% were other types of neurons and ~30% other brain cells.
Gating strategy	The cells were gated on a FSC-A vs SSC-A dot-plot with debris excluded. Single cells were gated on a FSC-W vs FSC-H plot. Parvalbumin positive cells were gated on an FSC-A vs. APC-A (PV AF647) plot. Cutoffs for parvalbumin positive and negative cells were determined based on FSC-A vs APC-A plots for unstained cells. Beta tubulin positive cells were gated on an FSC-A vs. FITC-A (Tub AF488) plot. Cutoffs for beta III Tubulin positive and negative cells were determined based on FSC-A vs FITC-A plots for unstained parvalbumin negative cells.

☒ Tick this box to confirm that a figure exemplifying the gating strategy is provided in the Supplementary Information.
