# Supplementary material for: LINE-1 retrotransposons contribute to mouse PV interneuron development
Source: Nat Neurosci. 2024 May 21;27(7):1274–84. doi: 10.1038/s41593-024-01650-2 (PMC11239520; doi:10.1038/s41593-024-01650-2)
Supplement: Supplementary file 6 — Uncropped gel image. [file 41593_2024_1650_MOESM6_ESM.pdf]

Uncropped gel image for Fig.1j.

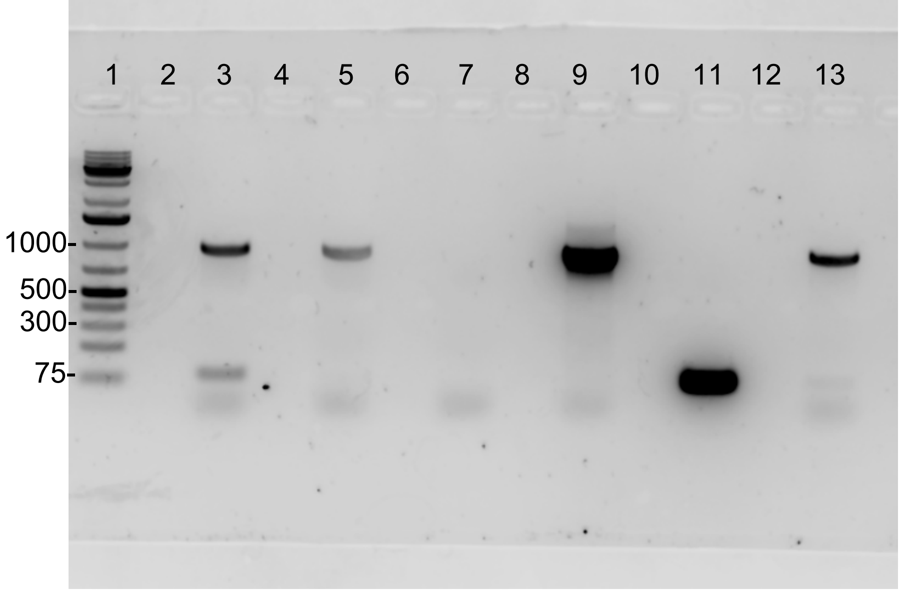

1- Molecular weight
2- Empty
3- $cL1_{spa}$ + Cre (DNA from electroporated primary neurons)
4- Empty
5- $cL1_{spa}$ no Cre (DNA from electroporated primary neurons)
6- Empty
7- Water
8- Empty
9- $L1_{spa}$ plasmid
10- Empty
11- mCherry plasmid
12- Empty
13- $cL1_{spa}$ RT^{-} + Cre (DNA from electroporated primary neurons)
