# Supplementary material for: LINE-1 retrotransposons contribute to mouse PV interneuron development
Source: Nat Neurosci. 2024 May 21;27(7):1274–84. doi: 10.1038/s41593-024-01650-2 (PMC11239520; doi:10.1038/s41593-024-01650-2)
Supplement: Supplementary file 11 — Uncropped gel image. [file 41593_2024_1650_MOESM11_ESM.pdf]

Uncropped gel image for Fig.5e.

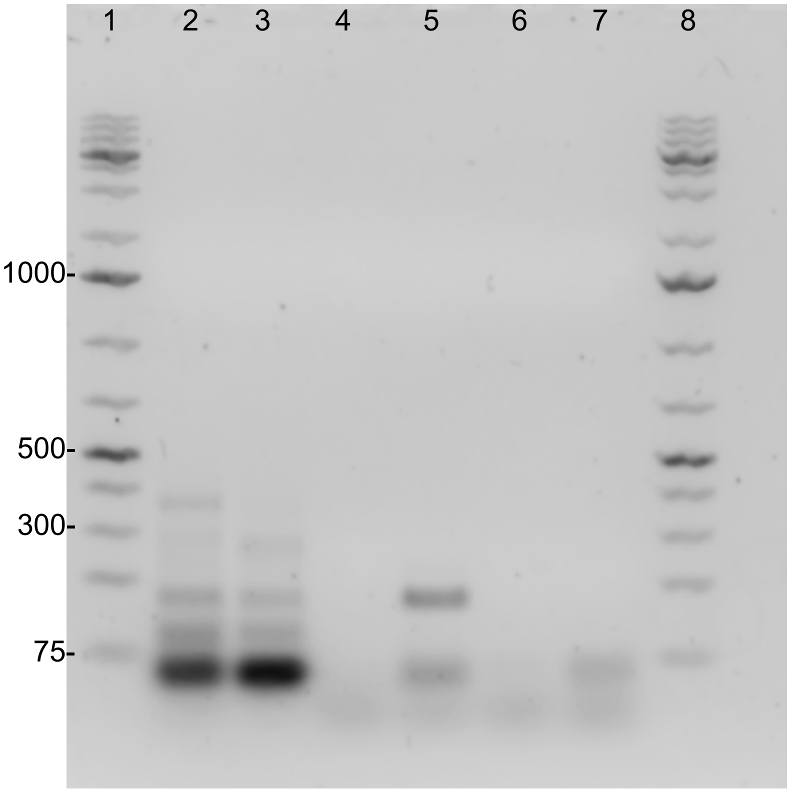

1- Molecular weight (bp)
2- Hippocampus (P35)
3- Hippocampus (P0)
4- PV- sorted cells
5- PV+ sorted cells
6- Water
7- RT-
8- Molecular weight (bp)
