# Supplementary material for: LINE-1 retrotransposons contribute to mouse PV interneuron development
Source: Nat Neurosci. 2024 May 21;27(7):1274–84. doi: 10.1038/s41593-024-01650-2 (PMC11239520; doi:10.1038/s41593-024-01650-2)
Supplement: Supplementary file 13 — Uncropped gel image. [file 41593_2024_1650_MOESM13_ESM.pdf]

Uncropped gel image for ExtendedFig.1a.

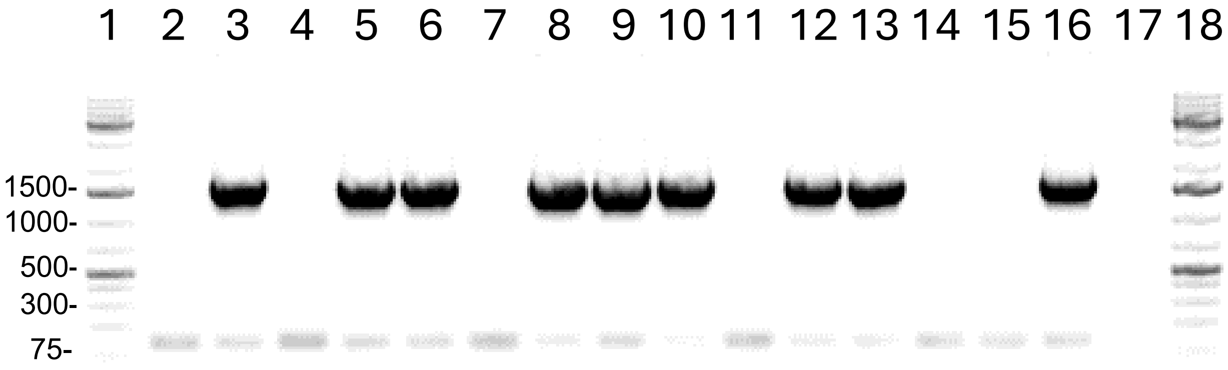

1- Molecular weight (bp)
2- Founder #1.2 x wt, offspring#1
3- Founder #1.2 x wt, offspring#2
4- Founder #1.2 x wt, offspring#3
5- Founder #1.2 x wt, offspring#4
6- Founder #1.3 x wt, offspring#1
7- Founder #1.3 x wt, offspring#2
8- Founder #1.3 x wt, offspring#3
9- Founder #1.3 x wt, offspring#4
10- Founder #1.3 x wt, offspring#5
11- Founder #1.3 x wt, offspring#6
12- Founder #8.1 x wt, offspring#1
13- Founder #8.1 x wt, offspring#2
14- Founder #8.1 x wt, offspring#3
15- Founder #8.1 x wt, offspring#4
16- L1-EGFP plasmid
17- Water
18- Molecular weight (bp)
